# The potential of *Paraburkholderia* species to enhance crop growth

**DOI:** 10.1007/s11274-025-04256-3

**Published:** 2025-02-05

**Authors:** Fernando Uriel Rojas-Rojas, Ingrid Melissa Gómez-Vázquez, Paulina Estrada-de los Santos, Harumi Shimada-Beltrán, Julio C. Vega-Arreguín

**Affiliations:** 1 Laboratorio de Ciencias AgroGenómicas, Escuela Nacional de Estudios Superiores Unidad León, Universidad Nacional Autónoma de México (ENES-León, UNAM), Blvd. UNAM 2011, 37684 León, Guanajuato México; 2 Laboratorio Nacional PlanTECC, Escuela Nacional de Estudios Superiores Unidad León, Universidad Nacional Autónoma de México (ENES-León, UNAM), Blvd. UNAM 2011, 37684 León, Guanajuato México; 3 Departamento de Microbiología, Escuela Nacional de Ciencias Biológicas, Instituto Politécnico Nacional, Prol. de Carpio y Plan de Ayala S/N Col. Santo Tomás Alc., 11340 Miguel Hidalgo, Ciudad de México México

**Keywords:** PGPR, Biocontrol, Crop, Biofertilizer

## Abstract

Agrochemicals are the primary alternative for maintaining the high yields necessary to produce sufficient plant-based foods to supply the world population. In recent decades, one of the most extensively explored alternatives to replace agrochemicals and reduce their environmental impact has been the use of microorganism-based products to boost crop yields with less environmental impact. This review focuses on the results of studies that have demonstrated the potential of the genus *Paraburkholderia* to increase crop yields and be utilized in biofertilizers and biocontrol products. A literature search was performed electronically considering articles and books published until August 19, 2024. We identified 24 species of *Paraburkholderia* with the ability to improve crop yields after their inoculation by different methods on seeds, seedlings, plantlets, adult crops, or fruits. The effects of these bacteria have been tested under laboratory, greenhouse, or field conditions. These *Paraburkholderia* species mediate their positive impact on crop growth by direct and indirect plant growth-promoting mechanisms, which include improving nutrient uptake, stimulating growth by phytohormone production, regulation and stimulation of metabolic pathways, induction of abiotic stress tolerance, and disease control by direct pathogen inhibition or induction of systemic resistance in plants. The literature reviewed here supports the use of *Paraburkholderia* in bio-inputs under the actual panorama of climate change and the necessity to increase sustainable agriculture worldwide.

## Introduction

The growing human population and the necessity to produce enough food to ensure food security for all people worldwide have led modern agriculture to rely on the use of inputs, such as chemical fertilizers, to enhance the productivity of crops and the quality of plant-derived foods. However, the excessive use of these products affects soil health by changing its quality, physicochemical properties, enzyme activity, compaction, acidity, microbiota, and salinity (Pahalvi et al. 2021). The use of biofertilizers is one of the most studied and promising alternatives to maintain the productivity of crops with low environmental impact (Pathania et al. 2020) and to reduce the overuse of chemicals to produce food through sustainable agricultural practices. These products have been applied worldwide to many crops, including tomato, wheat, and sugarcane, under field conditions in countries such as India, Italy, and Australia, respectively, showing positive effects on nutrient use efficiency, plant growth, and yield (Amaresan et al. 2019; Dal Cortivo et al. 2020; Qiu et al. 2022). Biofertilizers contain microorganisms whose inoculation in crop plants enhances seed germination, nutrient absorption, and growth; improves soil fertility; modulates plant metabolism; increases tolerance to biotic and abiotic stress; and helps in the bioremediation of contaminated soils (Basu et al. 2021). Despite the challenges in applying biofertilizers, such as their shelf life and the impact of climatic or geographical conditions (Orozco-Mosqueda et al. 2021), the development of plant growth-promoting bacteria (PGPB)-based products continues worldwide (Basu et al. 2021). These bacteria are commonly isolated from the rhizosphere or internal tissues of crop plants and can improve growth and yield through different plant growth-promoting (PGP) mechanisms. These mechanisms can act directly or indirectly on crop plants. Direct mechanisms include stimulation of growth by the production of phytohormones (auxins, cytokinins, and gibberellins), growth control by the enzyme 1-aminocyclopropane-1-carboxylate (ACC) deaminase, atmospheric nitrogen fixation, solubilization of phosphate and potassium to enhance their availability, and higher insoluble iron uptake by the production of siderophores. Control of plant pathogens that cause losses in crop production is an indirect way to promote growth and boost yield. PGPB can be considered biocontrol agents if they inhibit plant pathogens by producing antimicrobials, hydrogen cyanide, cell wall-degrading enzymes, nutrient competition, induction of systemic resistance in crop plants, and quenching of the quorum sensing phenomenon (Glick 2012; Olanrewaju et al. 2017). This review aims to compile current evidence to propose strains from *Paraburkholderia* as microorganisms suitable for developing biofertilizer products to increase crop yields.

### From *Burkholderia *s.l. to the PGPB-rich genus* Paraburkholderia*

The above-described PGP features have been reported in several bacteria, including genera from the β-proteobacteria group *Burkholderia* sensu lato s.l. (Barrera-Galicia et al. 2021). *Burkholderia* was described in 1992 (Yabuuchi et al. 1992) and for 22 years, was comprised of species recognized as pathogens of humans, plants, and animals, as well as environmental species with features to improve plant growth and remediate environments contaminated with different pollutants. Several studies have shown the potential of *Burkholderia* species to establish beneficial associations with plants (Suárez-Moreno et al. 2012). However, their phylogenetic relationship with pathogenic species and the lack of studies to discard pathogenicity restrict their use in agriculture and bioremediation. In 2014, a study conducted on symbiotic strains demonstrated the absence of virulence determinants and mammalian pathogenesis in environmental strains of *Paraburkholderia* (Angus et al. 2014). In the same year, a study using 16S rRNA gene sequences and 21 highly conserved proteins to identify conserved sequence insertions or deletions led to the description of the genus *Paraburkholderia* to re-classify 46 species of *Burkholderia,* comprising strains with PGP features, such as nitrogen fixation, and mainly isolated from environmental samples (Sawana et al. 2014). Owing to the taxonomic status of *Burkholderia* s.l., species from the original genus *Paraburkholderia* described in 2014 were re-classified into new genera. Currently, the *Burkholderia* s.l. group is composed of seven genera: *Burkholderia* sensu stricto (s.s.), *Paraburkholderia*, *Caballeronia*, *Robbsia*, *Mycetohabitants*, *Trinickia* and *Pararobbsia* (Lin et al. 2020). Among them, *Paraburkholderia* is the genus with the most significant number of species with potential applications in agrobiotechnology. Most strains of this genus have been isolated from soil, water, rhizosphere, internal tissues of plants, and legume nodules (Vio et al. 2020). The species of *Paraburkholderia* are currently identified based on traditional phenotypic and chemotaxonomic features, as well as genomics tools, including phylogenomics, phylogenetic analysis of concatenated conserved genes/proteins, average nucleotide identity (ANI) and digital DNA-DNA hybridization (dDDH) analysis (Beukes et al. 2017; Mullins and Mahenthiralingam 2021; Bach et al. 2023; Oh et al. 2024).

Vio et al. (2020) published an excellent book chapter on *Paraburkholderia* that included data from the literature up to January 2019 on the positive effects of *Paraburkholderia* species on plant growth. They provided evidence from in vivo experiments using crops and model plants grown in chambers, greenhouses, and fields under gnotobiotic conditions (Vio et al. 2020). Since then, more than 50 studies have been published on the effects of *Paraburkholderia* species on the growth parameters of different crops and model plants, such as *Arabidopsis thaliana* L. Heynh. and *Nicotiana benthamiana* Domin. Recent studies have investigated the ability of *Paraburkholderia* strains to modify and improve growth, metabolism, and stress tolerance of model plants (Wang et al. 2021; Macabuhay et al. 2022; Orellana et al. 2022; Shahzad et al. 2022).In this review, we have compiled the most relevant insights into the effects of *Paraburkholderia* species on crop growth, yield, and their PGP features.

**Fig. 1:**
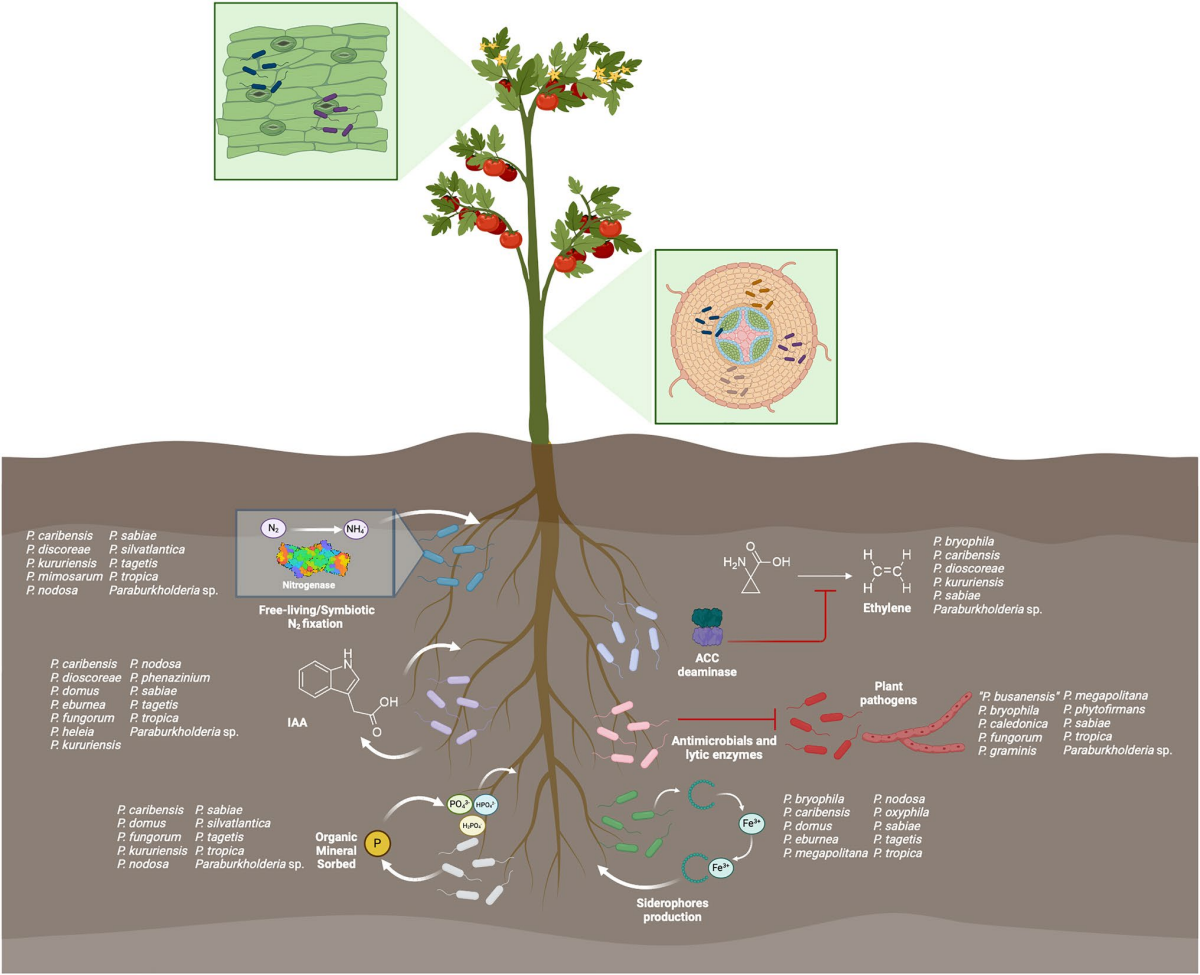
Colonization of *Paraburkholderia* species and plant-growth promoting mechanisms (PGP) reported to have the potential to enhance crop growth. The ability to colonize the internal tissues of crops, phyllosphere, and rhizosphere is represented. The PGP properties are indicated in the rhizosphere; however, some strains could express these properties on the internal tissues and phyllosphere of crops. Next to each mechanism, the producer species are listed. IAA indoleacetic acid, ACC 1-aminocyclopropane-1-carboxylate. Tomato was selected as a representative since more studies have been published on this crop. Created with BioRender.com

**Fig. 2:**
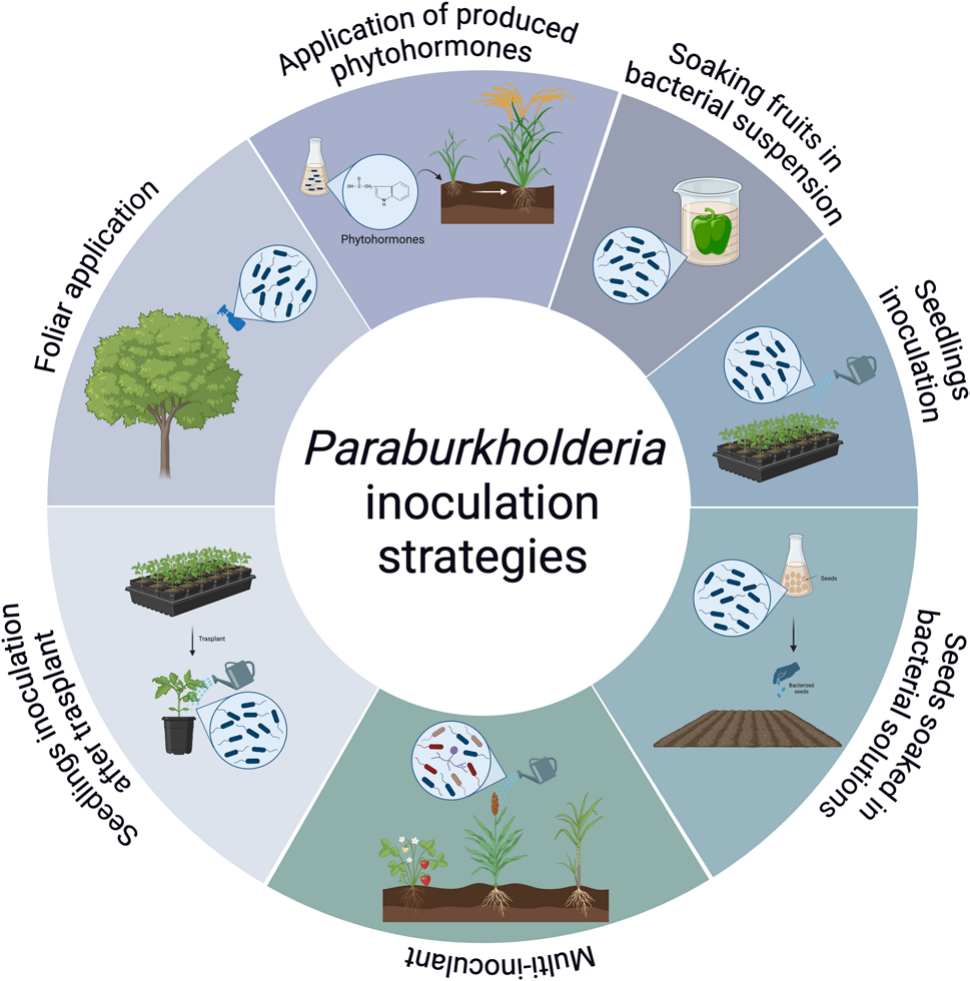
Inoculation strategies of *Paraburkholderia* species used in crops. The strains of different species of the genus *Paraburkholderia* have shown the ability to improve the growth of crops by inoculation through the strategies represented in the figure. Most of them include the use of one specific strain. However, inoculation has been tested in consortia with other microorganisms or phytohormones produced in culture media by these strains. Created with BioRender.com

**Fig. 3:**
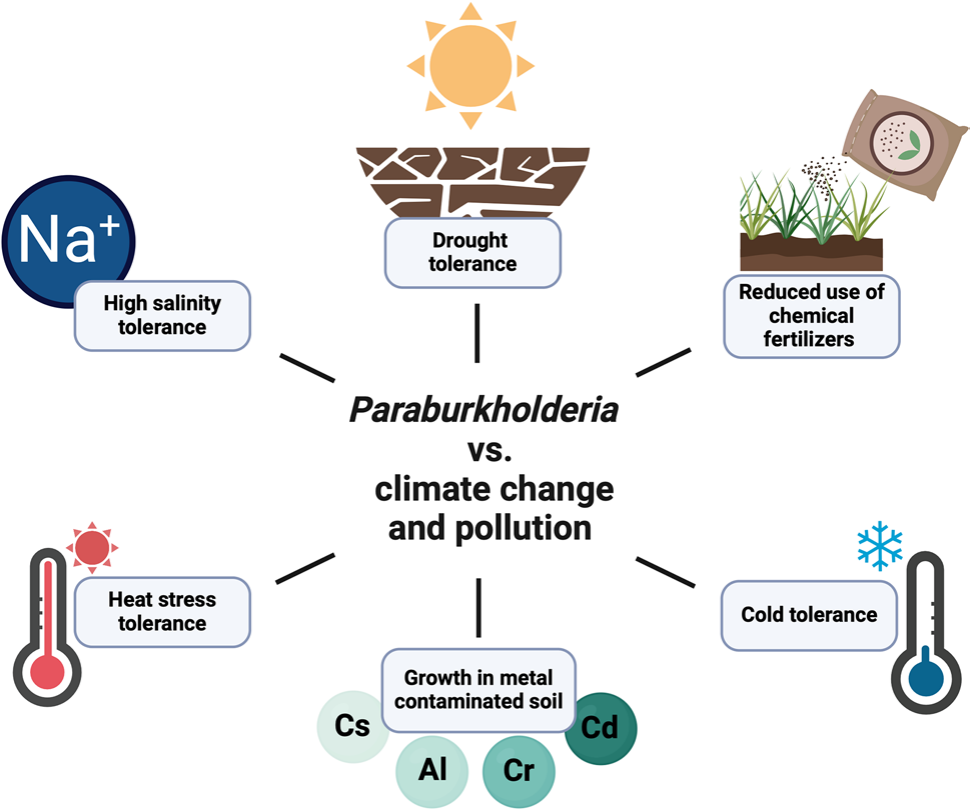
Benefits of *Paraburkholderia* inoculation on crops under climate change and chemical pollution scenarios. Positive effects of the inoculation of *Paraburkholderia* species on crops growing in abiotic stresses from climate change, chemical fertilizer use, and soil metal accumulation. Created with BioRender.com

The data presented here were obtained through a literature search mostly in Google Scholar, PubMed, and Science Direct databases until August 19, 2024, without language restrictions or a specific period. The keywords used included “*Paraburkholderia”*, “*Burkholderia”, “*plant growth promotion”, “PGPB”, “crop”, “yield” and “biocontrol”. Only reports that included experimental inoculation of seeds, seedlings, plantlets, fruits, or adult crop plants with *Paraburkholderia* strains were considered. The literature published before 2014, in which the actual *Paraburkholderia* species were named *Burkholderia,* was also included. Some authors continue to use *Burkholderia* for some *Paraburkholderia* species. In these cases, we confirmed that these species belong to *Paraburkholderia* and were included here. The percentages of the impact of *Paraburkholderia* strains on crop growth were obtained from the information provided by the authors or calculated when the article included numerical data. Literature in which only graphics were used to show these effects was considered, but the percentages were not calculated. PGP mechanisms were considered when experimentally demonstrated or related genes were identified through genome mining (Fig. 1). The most common inoculation strategies for the strains considered in this review are shown in Fig. 2. Additionally, we include strains with the potential to improve crop growth under climate change and pollution scenarios through different mechanisms (Fig. 3). The selected species are shown in three sections according to their potential to promote crop growth and their ability to control plant pathogens, such as bacteria, fungi, or both.

## Species for crop growth promotion

Some species *of Paraburkholderia* have shown the ability to promote the growth of different crops by increasing nutrient uptake, phytohormone production and modulation, and regulating plant genes related to growth (Table 1). However, no biocontrol ability has yet been reported. The effects of these species on crop development are described in detail in this section.

**Table 1:** Species of *Paraburkholderia* with the ability to promote crop growth

*Paraburkholderia* species	Strain (s)	Source	Tested crop	Effects on crop growth/yield	Plant growth-promoting mechanism(s) reported in the species (Direct and indirect)*	Reference
*P. caribensis*	XV	Mango (*Mangifera indica* L.) soil	Mango (*M. indica* cv "Ataulfo")	↑Roots, stems and leaves dry biomass, foliar area, floral fate, flowers number, foliar nitrogen, sugars content in leaves	Siderophore and IAA production, phosphate solubilization, nitrogen fixation and ACC deaminase activity Induction of the expression of genes involved in floral organs development, photosynthesis, sugar and nitrogen metabolism and mobilization Modulation of rhizosphere microbiome	de los Santos-Villalobos et al. (2013), Parra-Cota et al. (2014)
			Amaranth (*Amaranthus cruentu*s cv *Nutrisol* and *Amaranthus hypochondriacus* cv c*andil*)	↑Height, stem diameter, leaf area, foliar nitrogen, and total biomass (fresh and dry weight)		
	ISIB40	Sugarcane (*Saccharum officinarum* var. LP 85–384) rhizosphere	Soy bean (*Glycine max* L. Merr.)	↑Leaves number, shoot and root dry weight		De Gregorio et al. (2017)
	IAC-BECa-046	Sugarcane (*Saccharum officinarum* L.) roots	Sugarcane (*S. officinarum*)	↑ Shoot dry weight, root dry weight, total dry biomass, nitrogen amount and use efficiency index		da Silveira et al. (2019)
	IAC-BECa-088			↑Shoot and root biomass, accumulation and use efficiency of macro and micronutrients, photosynthesis and stomatal conductance		Marcos et al. (2015), Labanca et al. (2020), Cipriano et al. (2021), Leite et al. (2021)
	MZ956803	Lentil (*Lens culinaris* L.) rhizosphere	Lentil (*L. culinaris*)	↑Shoot weight, root length, nitrogen and phosphorus uptake and *Rhizobium* nodule number		Das et al. (2023)
*P. dioscoreae*	Msb3	Potato yam (*Dioscorea bulbifera* L.) leaves	Tomato (*Solanum lycopersicum* cv. Moneymaker)	↑Plant height, shoot length, shoot, root and total biomass	ACC deaminase activity and putative IAA production and nitrogen fixation	Herpell et al. (2020, 2021, 2023)
*P. domus*	VMSES32	Blueberry (*Vaccinium myrtillus* L.) roots	Blueberry (*V. myrtillus*)	↑ Root lenght	Phosphate solubilization and siderophores and IAA production	Gonçalves et al. (2022)
			Tomato (*Lycopersum esculentum* var. Roma)	↑Root length and secondary roots production		
*P. kururiensis*	KP23^T^	Aquifer environment	Rice (*Oryza sativa* L.)	↑Biomass, inflorescence and seeds	IAA production, putative ACC deaminase activity and nitrogen fixation Up-regulation of jasmonic acid-related genes Phosphate solubilization and ACC deaminase activity	Mattos et al. (2008), Dias et al. (2019)
			Maize (*Zea mays* L.)	↑Total biomass and root length		Alves et al. (2016)
	M130	Rice (*O. sativa*)	Rice (*O. sativa*)	↑Biomass and total nitrogen		Baldani et al. (1997), Divan-Baldani et al. (2000), Coutinho et al. (2013), Dias et al. (2019), Wallner et al. (2022), King et al. (2023)
			Maize (*Z. mays*)	↑Total biomass and root length		Alves et al. (2016)
	M130, 109, 120, M209, 16		Sorghum (*Sorghum bicolor* L.)	↑Biomass and total nitrogen		Dos Santos et al. (2017a, b)
	ATSB13	Rhizosphere soil	Canola (*Brassica campestris* L.)	↑Primary root length		Anandham et al. (2008)
*P. mimosarum*	LMG 23256^T^	*Mimosa pigra* L	Common bean (*Phaseolus vulgaris* L.)	↑Total shoot nitrogen content, nodules number and nodules dry weight	Nitrogen fixation	Chen et al. (2006), Lardi et al. (2017a, b)
*P. phenazinium*	CK-PC1	Hawai’i yellow-eyed grass (*Xyris complanate* R.Br.) rhizosphere	Hawaii yellow-eyed grass (*X. complanate*)	↑Roots and leaves length	Production of phenazine-1-carboxylic acid with auxin-like activity	Hane et al. (2021)
*P. phymatum*						
*P. silvatlantica*	UENF 117111	Pineapple (*Ananas comosus* L.)	Pineapple (*A. comosus*)	↑Biomass, phosphorus uptake, accumulation of nitrogen, phosphorus, potassium, calcium and magnesium ↑Fruit length, diameter, weight and sugar content	Nitrogen fixation and phosphate solubilization	Baldotto et al. (2010), Khuong et al. (2023)
	L-VT09 and N-VT06					Khuong et al. (2023)
	41, 3 m, 78 m, 85 m, 89 m and 90 m	Maize (*Z. mays*)	Maize (*Z. mays*)	↑Shoot and root biomass		Alves et al. (2016)
	PPCRr1, PPCRr3 and PPCRr8	Sugarcane (*S. officinarum*)	Sugarcane (*S. officinarum*)			
	41, 90 m, PPCRr-1 and PPCRr8	Maize (*Z. mays*) and Sugarcane (*S. officinarum*)	Sorghum (*S. bicolor*)			Dos Santos et al. (2017a, b)
*P. tagetis*	RG36^T^	Marigold roots	Tomato (*Solanum lycopersicum* L.)	↑Height, and fresh and dry weight of shoot and root	Phosphate solubilization, IAA and siderophores production and putative nitrogen fixation	Chhetri et al. (2023)

↑increased, ↓decreased, *IAA* indole-3-acetic acid, *ACC* 1-aminocyclopropane-1-carboxylic acid deaminase

*Effects on gene expression, metabolic pathways, and plant physiology were considered plant growth-promoting mechanisms

### *Paraburkholderia caribensis*

*Paraburkholderia caribensis* was first isolated from vertisol macroaggregates and was reported to be an exopolysaccharide-producing (Achouak et al. 1999) and haloacid-degrading species (Su et al. 2013). Strains of this species have also been isolated from interactions with plants, including wild legumes, mangoes, sugarcane, and lentils (Chen et al. 2003, 2005; de los Santos-Villalobos et al. 2013; Cipriano et al. 2021; Das et al. 2023). Although the first reports on the PGP of *P. caribensis* focused on nitrogen-fixing symbionts in *Mimosa diplotricha* and *Mimosa pudica* (Chen et al. 2005, 2003), positive effects on the growth of crops such as mango, amaranth, soybean, sugarcane, and lentils have been reported (de los Santos-Villalobos et al. 2013; Parra-Cota et al. 2014; De Gregorio et al. 2017; da Silveira et al. 2019; Cipriano et al. 2021; Das et al. 2023).

*P. caribensis* XV, isolated from mango soil, showed PGP properties in herbaceous and woody plants (de los Santos-Villalobos et al. 2013; Parra-Cota et al. 2014). This strain was used for microbial inoculation management treatment with *Rhizobium* sp. XXV on mango trees and induced biomass production in different tissues, including the foliar areas and roots, which increased by more than 50% (de los Santos-Villalobos et al. 2013). In amaranth plants, *P. caribensis* XV improved the total biomass (> 50%), significantly impacting leaves and stems (Parra-Cota et al. 2014). The PGP properties of this bacterium could act directly on nutrient assimilation by plants because an induced nitrogen uptake and expression of genes related to C4 photosynthesis and sugar mobilization in roots was observed.

Sugarcane can be considered an essential source for isolating *P. caribensis*. Strains IAC/BECa-046 and IAC/BECa-088 were isolated from the interaction with this crop. They showed the potential to improve growth by hormonal stimulation with indole acetic acid (IAA) production, thereby increasing shoot and root dry mass (da Silveira et al. 2019). Strain IAC/BECa-088 showed the best effects because these sugarcane growth parameters raised 50–90%, whereas the best parameter improved by IAC/BECa-046 was shoot dry mass, which increased by approximately 42%. Strain IAC/BECa-088 doubled the accumulation of macro-and micronutrients such as potassium, phosphorus, sulfur, zinc, and boron (Cipriano et al. 2021; Leite et al. 2021). Indirect PGP properties by modulating prokaryotic communities of the sugarcane rhizosphere and root endosphere have also been proposed for this strain, suggesting that *P. caribensis* has sufficient PGP properties to study their effects from the holobiont perspective (Leite et al. 2021). Strain IAC/BECa-088 was included in a multi-inoculant comprising bacteria from four different genera. However, this inoculant only improved sugarcane photosynthesis and stomatal conductance but did not improve the height, shoot, or root dry matter (Marcos et al. 2015). This consortium shows the importance of studying the interactions between bacteria used in multi-inoculants and the interactions between inoculated bacteria and native microorganisms in plants, as these interactions could modify the efficacy of bio-inoculants. Meristem culture sugarcane plantlets grown in acidic soil with low phosphate and potassium availability and high aluminum saturation were also inoculated with IAC/BECa 088 (Labanca et al. 2020). Expanded shoot biomass was observed with the accumulation of aluminum. No significant accumulation of micronutrients sufficient to mitigate aluminum-induced damage was observed, suggesting that this strain may not be an ideal choice as a PGPB for sugarcane cultivation in aluminum-contaminated acidic soils. This conclusion holds despite the observed effects on shoots and the positive results described in previous studies.

*P. caribensis* ISIB40 was also isolated from the sugarcane rhizosphere and retained its PGP properties after immobilization in polyvinyl alcohol nanofibers and successfully colonized soybean plants (De Gregorio et al. 2017). The nanofibers with strain ISIB40 increased leaf number and shoot dry weight, suggesting that incorporating bacteria in nanomaterials during bioproduct formulation could improve their shelf life, survival, and effectiveness in the field.

Most recently, the effects of *P. caribensis* have been reported in the grain legume lentil (Das et al. 2023). Strain MZ956803 was isolated from the lentil rhizosphere and produced IAA, HCN, siderophores, and solubilized phosphate. *In planta* experiments showed that *P. caribensis* MZ956803 increased shoot weight, root length, and phosphorus uptake. This strain is also compatible with different *Rhizobium* strains and induces nodule formation and nitrogen uptake in lentils when inoculated with this genus (Das et al. 2023). This influence of non-nodulating bacteria on legume nodule development has been proposed in different genera, including *Paraburkholderia* (Tapia-García et al. 2020).

Developing bio-inputs containing *P. caribensis* strains could be a sustainable option to improve sugarcane yield and reduce the use of chemical fertilizers for this crop. Furthermore, this species can retain its PGP properties in other crops, including legumes.

### *Paraburkholderia dioscoreae*

The recently described species *Paraburkholderia dioscoreae* includes the strains Msb3^T^ and Ch1-1 (Herpell et al. 2021). Msb3^T^ was isolated from the potato yam phyllosphere, it can colonize the tomato phyllosphere and promote growth when seedlings are exposed to the strain before transplantation (Herpell et al. 2020, 2023). Bacterized plants stood out for gaining more than 80% of their dry weight. The PGP attributes of this strain have yet to be tested in vitro. However, genome sequencing has revealed the presence of genes involved in nitrogen fixation, IAA production, and ACC deaminase activity. Recently, ACC deaminase was confirmed to be a critical PGP property of this bacterium in tomato and the model plant *Arabidopsis thaliana* (Herpell et al. 2023). A deficient ACC deaminase mutant showed a reduced ability to improve tomato growth, suggesting that the modulation of ethylene production is a crucial PGP trait in this phyllosphere bacterium. Since Msb3^T^ was isolated from a leaf surface of potato yam, the colonization of the tomato phyllosphere in bacterized seedlings suggests the ability of *P. dioscoreae* to adapt to different crops. These studies on *P. dioscoreae* Msb3^T^ open a new door for investigating the foliar application of *Paraburkholderia* strains and their positive effects on crop yield.

### *Paraburkholderia domus*

*Paraburkholderia domus* was described in 2021 as a species composed of strains isolated from forest, succulent, and industrial waste deposit soils (Vanwijnsberghe et al. 2021). Initially, this species was not recognized as a PGPB. However, in 2022, the strain VMSES31 was isolated from the root endosphere of blueberry (*Vaccinium myrtillus* L.) (Gonçalves et al. 2022). Among 142 isolates, VMSES31 showed the highest capacity to solubilize calcium and phosphate and produce siderophores and IAA (Gonçalves et al. 2022). This strain was inoculated on blueberry seedlings, inducing moderate root length (40%). Analysis of a transconjugant of VMSES31 containing a plasmid encoding a green fluorescent protein confirmed the ability of this strain to colonize the blueberry root surface and root epithelial cell unions. Because of the PGP properties observed in vitro and the good colonization pattern of the roots, this strain could modulate other blueberry growth parameters in long-term experiments.

Interestingly, the root length and production of secondary metabolites in tomato roots doubled in seedlings seven days post-inoculation with *P. domus* VMSES31, suggesting a better adaptation in this crop than in blueberry. The production of secondary metabolites in plant roots is a critical factor that regulates the presence of microorganisms in the rhizosphere; thus, *P. domus* VMSES31 could also modify the soil microbiome of tomato plants by modulating root secondary metabolism.

### *Paraburkholderia kururiensis*

*Paraburkholderia kururiensis* is a species of pollutant-degrading bacteria with the potential to remove hydrocarbons such as trichloroethylene, xylene, toluene, and benzene (Zhang et al. 2000; Ortega-González et al. 2013). Some strains have been isolated from polluted sites, whereas others are plant endophytes (Zhang et al. 2000; Mattos et al. 2008; Coutinho et al. 2013; Ortega-González et al. 2013; Wallner et al. 2022). However, environmental and endophytic strains have demonstrated PGP features in crops.

*P. kururiensis* KP23^T^ is a pollutant-degrading bacterium that can colonize roots and aerial parts of rice plants and induces 9- to 10-fold growth in root biomass, 5- to 6-fold rise in aerial biomass, and 4- to 5-fold enhancement in inflorescence and seed counts (Mattos et al. 2008). The KP23^T^ genome contains genes associated with PGP features such as ACC deaminase, IAA biosynthesis, and nitrogen fixation (Dias et al. 2019). IAA produced by KP23^T^ in culture media stimulates the formation of additional lateral roots and root hairs (Mattos et al. 2008). Therefore, this bacterium can be applied directly as an inoculant or utilized as a source of metabolites to promote rice growth.

The rice endophyte *P. kururiensis* M130 is the most studied strain of this species in terms of its PGP features. This bacterium is a root endophyte found in rice (Baldani et al. 1997; Wallner et al. 2022), which slightly increases biomass production and total nitrogen (Divan Baldani et al. 2000). The M130 geno3me includes genes related to direct PGP features such as ACC deaminase, IAA biosynthesis, and nitrogen fixation (Coutinho et al. 2013; Dias et al. 2019). However, after root colonization, the strain M130 upregulates jasmonic acid-related genes (King et al. 2019), which could indirectly promote rice growth by inducing resistance to biotic and abiotic stresses. This bacterium is an excellent candidate for inclusion in rice inoculants to improve development and health.

Strains KP23^T^ and M130 are strong candidates for enhancing maize growth. These strains, along with three other strains, have been shown to significantly increase root (400–500%) and shoot biomass (20–50%) (Alves et al. 2016). However, the molecular interactions between *P. kururiensis* and maize have not yet been characterized. Nitrogen fertilization is crucial for the successful cultivation of maize, and the use of diazotrophic bacteria is a suitable alternative to diminish the application of inorganic nitrogen fertilizers (Kifle and Laing 2016); *P. kururiensis* strains are promising inoculants for improving maize growth and increasing nitrogen uptake because of their ability to fix nitrogen.

*P. kururiensis* strains also showed the potential to enhance sorghum growth in the greenhouse (dos Santos et al. 2017a, b). Five strains influenced biomass production in this crop, two of which were included in a multi-inoculant that enhanced biomass and total nitrogen in sorghum plants.

Another strain with a single analysis of its PGP properties is *P. kururiensis* ATSB13, which was isolated from rhizosphere soil in Korea (Anandham et al. 2008). This bacterium grows in a nitrogen-free culture medium, solubilizes phosphate, produces ACC deaminase, and increases primary root length by 154.1% in canola seedlings (Anandham et al. 2008).

Given the pollutant-degrading capability of *P. kururiensis* strains and their PGP features demonstrated in rice and sorghum, this genus serve as a valuable source of bacteria for enhancing the growth and yield of monocots, even in contaminated soils.

### *Paraburkholderia mimosarum* and *Paraburkholderia phymatum*

Nodulation is a symbiotic interaction between legume plants and bacteria that leads to the formation of a specialized plant organ known as a root nodule. Within these nodules, bacteria convert atmospheric dinitrogen into ammonia, a form of nitrogen that plants can assimilate. The most studied nodulating bacteria belong to the subclass α-Proteobacteria, as well as the genera *Paraburkholderia* and *Cupriavidus* from the subclass β-Proteobacteria (Hassen et al. 2020). The nodulating *Paraburkholderia* species include *P. caribensis*, *Paraburkholderia diazotrophica*, *Paraburkholderia mimosarum*, *Paraburkholderia nodosa*, *Paraburkholderia phenoliruptrix*, *Paraburkholderia phymatum*, *Paraburkholderia sabiae*, *Paraburkholderia symbiotica* and *Paraburkholderia tuberum,* which are commonly isolated wild legumes and can nodulate other wild legumes in addition to their original hosts (Chen et al. 2005; Elliott et al. 2009; Liu et al. 2011; Mishra et al. 2012; Bournaud et al. 2013; Tapia-García et al. 2020). Derived from host specificity and the lack of agricultural importance of wild legumes, little is known about the potential of *Paraburkholderia* species to form nodules and improve nitrogen acquisition and growth of crop legumes. Some species have shown positive effects on the growth of non-legume crops; we discuss these species in other sections of this review. However, *P. mimosarum* and *P. phymatum* have demonstrated the ability to form nodules in bean (*Phaseolus vulgaris* L.) (Lardi et al. 2017a, b; Liu et al. 2020; Talbi et al. 2010), which is one of the most important crop legumes worldwide.

*P. mimosarum* is mainly isolated from nodules of *Mimosa* species in South America and Asia (Chen et al. 2006; Elliott et al. 2009; Liu et al. 2011). This species is a highly competitive symbiont in this legume genus (Elliott et al. 2009). Interestingly, inoculation with *P. mimosarum* LMG 23256^T^ induced nodules in *P. vulgaris* and improved nutrient accumulation, causing a double content of total nitrogen in the shoot (Lardi et al. 2017b). *P. phymatum* was originally isolated from the wild legume *Machaerium lunatum* (Vandamme et al. 2007), but it is well known for its ability to form nitrogen-fixing nodules in bean (Talbi et al. 2010; Liu et al. 2020). Unfortunately, the studies on the symbiotic interaction between *P. phymatum* and beans have not reported improvement in parameters related to crop yield.

*P. vulgaris* L. has been used as a trap plant to isolate nodulating bacteria from the soil of wild legumes, including *Paraburkholderia* strains (Dall’Agnol et al. 2016; Tapia-García et al. 2020). This indicates the ability of nodulating *Paraburkholderia* to form a symbiotic interaction with this crop, even when the original host is a wild legume. Thus, studies on the symbiotic interaction between nodulating species such as *P. mimosarum* and *P. phymatum* and bean should pay more attention to their impact on yield parameters.

### *Paraburkholderia phenazinium*

*Paraburkholderia phenazinium* was first described in 1973 as a *Pseudomonas* species (Bell and Turner 1973). Despite the taxonomic reassessment of *Burkholderia* and *Paraburkholderia,* there have been few studies on this species, with most focusing on its production of antimicrobial phenazine pigments (Byng and Turner 1977; Messenger and Turner 1983). Recently, the strain CK-PC1 of *P. phenazinium* was isolated from the soil in the Kalampangan area of Indonesia (Hane et al. 2021). This soil promoted the germination and growth of Hawaii yellow-eyed grass (*Xyris complanate* R.Br.). Co-inoculation of *Penicillium rolfsii* Y-1 and *P. phenazinium* CK-PC1 in the seeds of this plant promoted root and leaf development, suggesting that co-inoculation with *Paraburkholderia* strains and fungi could be an alternative for developing new bioinoculant products based on different microorganisms. Additionally, a positive effect on seedling growth was observed when seedlings were treated with phenazine-1-carboxylic acid (PCA) produced by strain CK-PC1, which demonstrated auxin-like activity similar to IAA in *Vigna radiata* (L.) R.Wilczek (Hane et al. 2021). Given the PGP properties of the strain CK-PC1 and the well-characterized antimicrobial activity of the phenazine pigments, which include the inhibition of plant pathogens such as *Pythium ultimum*, *Rhizoctonia solani*, and *Fusarium oxysporum* (Yan et al. 2021), this species shows potential for developing bio-inputs aimed at sustainable control of plant diseases caused by fungal pathogens.

### *Paraburkholderia silvatlantica*

Another species with the potential to formulate bio-inoculants for monocots is *Paraburkholderia silvatlantica.* This species was first described in 2006 to classify the diazotrophic bacteria associated with maize, sugarcane, and pineapple (Perin et al. 2006). In pineapple, strains of this species induced biomass production (100%), duplicated the accumulation of nitrogen, phosphorus, and calcium, and slightly increased fruit length, fruit diameter, weight per fruit, and fruit sugar (Baldotto et al. 2010; Khuong et al. 2023). In maize and sorghum, shoot and root biomass increased five- and two-fold, respectively, because of the inoculation of different diazotrophic *P. silvatlantica* strains (Alves et al. 2016). Unfortunately, beyond the inoculation of maize, pineapple, and sorghum, only the ability of *P. silvatlantica* to fix nitrogen and solubilize phosphate has been studied. Further studies could explore additional PGP features and the molecular interactions of this species with monocots.

### *Paraburkholderia tagetis*

*Paraburkholderia tagetis* is one of the most recently described species of the genus and is only composed of the strain RG36^T^ isolated from *Tagetas patula* roots (Chhetri et al. 2023). Tomato seedlings inoculated with *P. tagetis* RG36^T^ showed increased shoot (20%) and root (79%) length, shoot (64%) and root (73%) biomass, and fruit yield (> 300%). This strain exhibited in vitro PGP properties, including IAA and siderophores, phosphate solubilization, nitrogen fixation, and putative genes related to these activities (Chhetri et al. 2023). The study of the effects of *P. tagetis* on tomato showed that strains isolated from no-crop plants could be helpful for sustainable agricultural production, similar to the discussion for *Paraburkholderia* species isolated from wild legumes.

## Species for growth promotion and biocontrol of bacterial diseases

In addition to their ability to promote crop growth, the three species described next have the potential to control phytopathogenic bacteria and to be used in biocontrol products (Table 2).

**Table 2:** *Paraburkholderia* species with the potential to promote crop growth and control bacterial diseases

*Paraburkholderia* species	Strain (s)	Source	Tested crop	Effects on crop growth/yield	Plant growth-promoting mechanism(s) reported in the species (Direct and indirect)	Reference
*P. heleia*	PAK1-2	Rice (*Oryza sativa* var. j*aponica* cv. Koshihikari)	Rice (*O. sativa* var. *japonica* cv. Koshihikari)	↑ Root length ↓ Symptoms of blight in rice seedlings	IAA production	Wang et al. (2016)
*P. nodosa*	CNPSo 1258 and CNPSo 1294	Wild legume soil	Common bean (*Phaseolus vulgaris* L.)	↑ Shoot dry weight, nitrogen concentration and total nitrogen	Competition for nutrients, nitrogen fixation, IAA production, phosphate solubilization and siderophore production	Dall’Agnol et al. (2016)
	CNPo 3076	*Mimosa micropteris* Benth. nodules	Maize (*Zea mays* L.)	↑ Fresh and dry root, shoot and total weight in half phosphate fertilization		Nascimento et al. (2024)
	CNPo 3281	*Mimosa paranapiacabae* Barneby nodules				
	G5.2rif1	Burn forest	Tomato planlets (*Lycopersocum esculentum* cv. Momotaro)	↑ Yield ↓ Bacterial wilt incidence		Nion and Toyota (2008)
	NB1	Soil from a rehabilitated forest	Maize (*Z. Mays*)	↑ Leaves and root dry weight, height, stem diameter, total biomass, chlorophyll content, as well as leaves, stem and roots nitrogen concentration		Tang et al. (2020a, b)
			Green gram *Vigna radiata* (L.) R.Wilczek	↑ Shoot length and seedling vigor index		
*P. sabiae*	LMG 24235^T^	*Mimosa caesalpiniifolia* Benth. nodules	Potato (*Solanum tuberosum* L.)	↓ Symptoms of the soft root disease	Phosphate and potassium solubilization, IAA and siderophores production, ACC deaminase activity and putative nitrogen fixation In vitro antagonism of *Burkholderia gladioli*, *Burkholderia plantarii*, *Burkholderia glumae*, *Ralstonia solanacearum*, *Pseudomonas syringae*, *Pectobacterium carotovorum* and *Erwinia amylovora*	Hug et al. (2023)
	MG774427	Soybean (*Glycine max* L. Merr.) rhizosphere	Kamatsuna (*Brassica rapa* var. *Perviridis*)	↑ Shoot biomass and ^137^Cesium isotope uptake		Rallos et al. (2021)
	NvS21	Forest soil	Soybean (*G. max*)	↑ Shoot length, total dry weight, number of nodules per plant, nodule fresh and dry weight, chlorophyll content, grain yield and nitrogen, phosphorus and potassium (NPK) uptake		Solanki et al. (2023)
			Chickpea (*Cicer arietinum* L.)	↑ Shoot and root length and total dry weight		

↑Increased, ↓Decreased, *IAA* indole-3-acetic acid, *ACC* 1-aminocyclopropane-1-carboxylic acid deaminase

### *Paraburkholderia heleia*

*Paraburkholderia heleia* is a poorly studied species. The first report described three strains isolated from aquatic plants, with limited investigation of their PGP properties beyond nitrogen fixation (Aizawa et al. 2010). More recently, *P. heleia* PAK1-2 was isolated from the rice rhizosphere and suggested as a PGPR and a biocontrol agent for the plant pathogen *Burkholderia plantarii* (Wang et al. 2013, 2016). Rice seeds soaked in a PAK1-2 suspension developed longer roots than non-inoculated seeds. In dual-culture experiments, *P. heleia* PAK1-2 alleviated the poor growth of stems and roots associated with blight caused by *B. plantarii.* The biocontrol activity of *P. heleia* was related to the production of IAA, which interfered with the biosynthesis of tropolone, a phytotoxin required to induce blight disease in rice seedlings caused by *B. plantarii* (Wang et al. 2016). Microorganisms of aquatic plants have been proposed as potential biocontrol agents with PGP features, including IAA production (Pramanic et al. 2023). Since this is the main PGP property of PAK1-2, similar studies on *P. heleia* strains isolated from aquatic plants could support the development of bio-input based on their IAA production and biocontrol features.

### *Paraburkholderia nodosa*

*Paraburkholderia nodosa* is another nodulating species comprising strains isolated from nodules and soil of wild legumes native to Brazil (Chen et al. 2007; Dall’Agnol et al. 2016, 2017; Paulitsch et al. 2019). Some strains can nodulate wild legumes in addition to their original hosts (Chen et al. 2007; Dall’Agnol et al. 2017), and common bean (*P. vulgaris*) is often used as a trap plant for isolation (Dall’Agnol et al. 2016, 2017). Strains CNPSo 1258 and CNPSo 1294, isolated from wild legume soils, increased shoot dry weight (2- and 3-fold), nitrogen concentration (fourfold), and total nitrogen content (4- and 12-fold) in *P. vulgaris* (Dall’Agnol et al. 2016). The effects of other strains isolated from wild legumes on the growth parameters of common bean remain unknown. Unlike the nodulating species *P. mimosarum* and *P. phymatrum* discussed above, the PGP properties of *P. nodosa* have been documented in non-legume crops. Interestingly, strains exhibiting these effects were isolated from non-legume soils outside South America (Nion and Toyota 2008; Tang et al. 2020a).

*P. nodosa* G5.2rif1 was isolated from the soil of a burn forest in Kalimantan, Indonesia (Nion and Toyota 2008). This strain helps prevent losses in tomato yield caused by bacterial wilt (*Ralstonia solanacearum*) and reduces the incidence of the disease by 32–71% (Nion and Toyota 2008). Although strain G5.2rif1 did not exhibit in vitro antibiosis against plant pathogens, its suppressive effect is believed to result from its high competitiveness for nutrients when using different carbon and nitrogen sources.

*P. nodosa* NB1 was isolated from the rhizosphere of a tree in a rehabilitated forest in Malaysia (Tang et al. 2020b). This strain exhibited free-living nitrogen fixation, phosphate solubilization, and weak IAA production. However, inoculation of green gram (*Vigna radiata*) seeds with NB1 increased shoot length and seedling vigor index by approximately 42% after one week of growth (Tang et al. 2020b). In maize, the inoculation of NB1 in the soil at 50% of the total fertilization requirement enhanced some plant growth parameters, most notably increasing nitrogen concentration in leaves (52%), stems (33%), and roots (35%) (Tang et al. 2020a). These results were comparable with those obtained with 100% chemical fertilization and a treatment combining *P. nodosa* NB1 with a cellulolytic strain of *Serratia nematodiphila*, highlighting the high efficacy of *P. nodosa* NB1 as a biofertilizer that can reduce the need for chemical fertilizers and its compatibility with other PGPB. Other strains that can help reduce the use of chemical fertilizers are *P. nodosa* CNPSo 3281 and CNPSo 3076, both isolated from wild legumes of the genus *Mimosa* (Nascimento et al. 2024). These strains were inoculated into maize plants fertilized with half of the recommended triple superphosphate dose. Inoculation with *P. nodosa* CNPSo 3281 led to a 62.5% increase in fresh weight and a 52.6% increase in dry total weight, and inoculation with *P. nodosa* CNPSo 3076 maintained yields similar to those obtained with the full recommended fertilizer dose. These strains are known to be phosphate-solubilizing and siderophore-producing bacteria, which likely explains their ability to enhance nutrient acquisition in maize to improve nutrients, even with reduced chemical fertilization.

The positive effects of *P. nodosa* strains from Asia on maize and tomato suggest that bean-nodulating strains isolated from wild legumes in South America could also be explored to assess their potential as bio-inputs for both legume and non-legume crops. This research further supports the idea that this species comprises wild legume nodulating bacteria that are capable of improving crop yields.

### *Paraburkholderia sabiae*

*Paraburkholderia sabiae* is a legume-associated species isolated from nitrogen-fixing nodules of *Mimosa caesalpiniifolia* (Chen et al. 2008) and the nodules and soil of wild and crop legumes (Dall’Agnol et al. 2017; Silva et al. 2018; Rallos et al. 2021). The positive effects of this species on legumes and non-legume crops include both direct and indirect PGP mechanisms.

The type strain of *P. sabiae* LMG 24235^T^ is a promising biocontrol agent for bacterial diseases. This strain antagonizes in vitro the plant pathogens *Burkholderia gladioli*, *B. plantarii*, *Burkholderia glumae*, *R. solanacearum*, *Pseudomonas syringae*, *Pectobacterium carotovorum* and *Erwinia amylovora* (Hug et al. 2023). *In planta*, *P. sabiae* LMG 24235^T^ decreased the symptoms of soft root disease caused by *P. carotovorum* in potato tubers, confirming its potential as a biocontrol agent for bacterial diseases.

The bioremediation of metals is another possible application of *P. sabiae* strains. Radiocesium (^137^Cs) is a cesium isotope that poses a risk to animal and human health when incorporated into cells via the food chain (Rai and Kawabata 2020). This metal shares chemical properties similar to those reported for potassium and can be taken up by plants from contaminated soils (Rai and Kawabata 2020).Thus, isotope-accumulating plants could improve bioremediation in these soils. The potassium-solubilizing strain *P. sabiae* MG774427, isolated from the soybean rhizosphere, increased the accumulation of ^37^Cs in komatsuna (*Brassica rapa* L. var. *perviridis*) growing in contaminated soil (Rallos et al. 2021). *P. sabiae* MG774427 also induces shoot, root, and total biomass of komatsuna (Rallos et al. 2021), suggesting that this strain could help accumulative plants in the bioremediation of radioactive metals.

Similar to other *Paraburkholderia* species, *P. sabiae* strains isolated from forest environments can enhance crop growth. *P. sabiae* NvS21 isolated from forest soil, exhibits in vitro PGP properties, including phosphate solubilization, IAA and siderophores production, ACC deaminase activity, nitrogen fixation, and improved soybean and chickpea growth (Solanki et al. 2023). In the case of chickpea, inoculated seeds improved growth parameters, highlighting the shoot length (101%). In soybean, the strain slightly increased shoot length (12%) and dry weight (14%). Furthermore, the influence of NvS21 on soybean was determined by a field experiment of 60 days. Plants from bacterized seeds produced more nodules with larger fresh and dry weights and showed gains in chlorophyll A and B content, grain yield, and uptake of nitrogen, phosphorus, and potassium (Solanki et al. 2023). *P. sabiae* NvS21 could be also included in bio-inputs made of bacterial consortia to improve soybean yields since the co-inoculation with *Bacillus subtilis* in this legume resulted in improved nodulation, photosynthetic pigments content, straw yield, and grain NPK content (Solanki et al. 2023). Developing biofertilizers based on different microbes could be a more efficient alternative to promote crop yields because, most of the time, a single strain cannot improve all crop yield parameters and *P. sabiae* NvS21 is a good candidate for developing these products.

Finally, *P. sabie* is suitable for enriching crop growth under abiotic stress. Some isolates from wild legumes show tolerance to salt and heat stress and grow on benzene as the sole carbon source (de León-Martínez et al. 2017), suggesting a putative use of this species to mitigate the effects of climate change on crop yields. However, field evidence needs to be collected for these bacteria to develop a deeper understanding of this issue.

## Species for growth promotion and biocontrol of fungal diseases

Fungal diseases are among the most studied biotic stresses affecting crop production worldwide. For this reason, antagonistic microorganisms are continuously examined to develop biocontrol products to combat these pathogens sustainably. This potential has also been observed in some *Paraburkholderia* species and strains identified as *Paraburkholderia* sp. (Table 3), which also improve crop growth through direct PGP mechanisms.

**Table 3:** *Paraburkholderia* species with the potential to promote crop growth and control fungal diseases

*Paraburkholderia* species	Strain (s)	Source	Tested crop	Effects on crop growth/yield	Plant growth-promoting mechanism(s) reported in the species (Direct and indirect)*	Reference
*“P. busanensis”*	P39	Pine forests soil	Green pepper (*Capsicum annuum* cv. Nokwang)	Suppression of anthracnose in fruits	Production of antifungal compounds and inhibition of *Colletotrichum scovillei*	Mannaa et al. (2023)
*P. caledonica*	No indicated	Soil from Atacama Desert	Strawberry (*Fragaria vesca* cv. Alexandria)	↑ Biomass, fruit number, K^+^ concentration, antioxidant activity, chlorophyll content and photosynthetic parameters	In vitro inhibition of the pathogen *Rhizoctonia solani* by volatile compounds	Pérez-Moncada et al. (2024)
*P. eburnea*	CS4-2	Japonica rice (*Oryza sativa* cv. Dongjin)	Japonica rice (*O. sativa* cv. Dongjin)	↑ Root length and shoot weight	Silicate solubilization, siderophores and IAA production, induction of systemic resistance and presence of genes related to metallophores and antibiotics production	Kang et al. (2017)
	EP3	Eggplant (*Solanum melongena* cv. Black beauty) soil	Eggplant (*S. melongena* cv. Black beauty)	↓ Severity and incidence of disease caused by *Verticillium dahliae*		Poulaki et al. (2024)
*P. fungorum*	BRRh-4	Rice (*Oryza sativa* L.) rhizosphere	Rice (*O. sativa*)	↑ Seed germination, shoot and root length, shoot fresh and dry weight, root fresh dry weight, and grain yield	Phosphate solubilization and IAA production. Prevention of oxidative stress Induction of the activity of antioxidant enzymes Modulation of root and rhizosphere microbiome. Inhibition of plant pathogens (*Botrytis cinerea, Fusarium oxysporum, Nigrospora oryzae, R. solani* and *Alternaria alternata*)	Khan et al. (2017), Islam et al. (2023)
			Strawberry (*Fragaria ananassa* Duchesne)	↑ Shoot and root fresh/dry weight, canopy diameter, and leaf length, width and number ↑ Content of anthocyanins, carotenoids, flavonoids and phenolic contents		Rahman et al. (2018)
			Rapeseed (*Brassica campestris* cv. BARI Sarisha-14)	↑ Height, root fresh and dry biomass, and shoot fresh and dry biomass in the presence of toxic concentrations of cadmium		Raihan et al. (2022)
	PSB7	Barley (*Hordeum vulgare* L.) ectorhizosphere	Barley (*H. vulgare*)	↑ Phosphate assimilation, dry weight and height		Ibáñez et al. (2021)
*P. oxyphila*	EP1, EP 4 and EP5	Eggplant (*S. melongena* cv. Black beauty) soil	Eggplant (*S. melongena* cv. Black beauty)	↓ Severity and incidence of disease caused by *V. dahliae*	Siderophores production and presence of genes involved in metallophores and antibiotics production	Poulaki et al. (2024)
*P. tropica*	MTo-431	Maize (*Zea mays* L.) rhizosphere	Maize (*Z. mays*)	Favorable plants growth and absence of fungal growth in plants inoculated with a mixture of plant pathogens and MTo431	Inhibition of plant pathogens (*Ceratocystis paradoxa*, *Colletotrichum gloesporioides, Fusarium culmorum, F. oxysporum, Fusarium verticillioides* and *Sclerotium rolfsii*) by VOCs production Nitrogen fixation, phosphate solubilization and siderophore and IAA production	Tenorio-Salgado et al. (2013)
	PPe8^T^	Sugarcane (*Saccharum officinarum* L.) stem	Sugarcane (*S. officinarum*)	↑ Roots and shoot dry mass of seedlings, total nitrogen, potassium, and phosphorus of shoot and roots, total potassium of roots, nitrate reductase in root planlets, and improved germination speed ↑ Stalk and sugar yields (Ppe8^T^-containing multi-inoculant)		Chaves et al. (2015), Girío et al. (2015), dos Santos et al. (2017a, b, 2019), Pereira et al. (2019)
	MTo-293	Maize (*Z. mays*) stem	Tomato (*Lycopersicum esculentum* Mill.)	↑ Number and weight of fruits. Higher root and aerial part dry weight		Bernabeu et al. (2015), Vio et al. (2022)
	IAC/BECa 135	Sugarcane (*S. officinarum*) roots	Sorghum (*Sorghum bicolor* L.)	↑ Root dry weight and specific density and shoot biomass		Schlemper et al. (2018), Kuramae et al. (2020), Labanca et al. (2020), Cipriano et al. (2021), Leite et al. (2021)
			Sugarcane (*S. officinarum*)	↑ Shoot and root biomass, and improved accumulation, use efficiency of macro and micronutrients ↑ Shoot biomass under aluminum toxic concentration in soil		
	Ppe5, Ppe6, Ppe7	Sugarcane (*S. officinarum*)	Sorghum (*S. bicolor*)	↑ Biomass		dos Santos et al. (2017a, b), Schlemper et al. (2018)
*Paraburkholderia* sp.	NhPBG1	*Nepenthes hamblack* pitchers	Ginger (*Zingiber officinale* Roscoe)	Inhibition of *Pythium aphanidermatum* growth in rhizome	In vitro inhibition of the pathogens *P. aphanidermatum*, *R. solani*, *F. oxysporum* and *Colletotrichum accutatum* Production of antimicrobial compounds IAA and ammonia production, phosphate, potassium and zinc solubilization, nitrogen fixation and ACC deaminase activity	Ravi et al. (2021)
	B25	Maize (*Z. maize*) rhizosphere	Barley (*H. vulgare*)	Protection of Barley leaves against the net blotch diseases ↓ Negative impact net blotch on photosynthesis	Antagonism of the fungus *Drechslera teres*, the causal agent of net blotch	Backes et al. (2021a, b)
	ESA 688	*Vigna radiata* L. nodules	Cowpea (*Vigna unguiculata* L. Walp.)	↑ Emergence of seeds and protection of *Fusarium* sp. in seed emergence	Inhibition of *Fusarium* sp. growth in vivo	da Silva et al. (2021)
	ESA 700					
	P12	Blueberry (*Vaccinium myrtillus* L.) rhizosphere	Blueberry (*V. myrtillus*)	↑ Seed germination rate	Inorganic phosphate and silicate solubilization, auxins production and nitrogen fixation	Wang et al. (2022)
	P17					
	SOS3	No specified	Sugarcane (*S. officinarum*)	↑ Weight, sett, number and sucrose yield of sugarcane with organic and inorganic fertilizers ↑ Height, till number, cane and sucrose yield in no bacterized ratoons with organic and inorganic fertilizers	Antagonism against fungal pathogens such as *P. oryza*e, *R. solani* and* H. oryzae* Enhancement of nitrogen uptake	Gallart et al. (2021), Kanjanasopa et al. (2021), Paungfoo-Lonhienne et al. (2020, 2021), Petersen et al. (2021), Gallart et al. (2022)
			Rice (*O. sativa*)	↑ Germination ↓ Symptoms of diseases caused by *R. solani*, *Pyricularia oryzae* and *Helminthosporium oryzae*		
			Sorghum (*S. bicolor*)	↑ Shoot and root dry biomass and vigour index		
			Maize (*Z. mays*)	↑ Emergence percentage, leaf number, height, shoot dry biomass and vigour index		
			Avocado (*Persea americana* Mill.)	↑ Nitrogen uptake		
			*Macadamia integrifolia* Maiden and Betch	↑ Leaf, stem and root dry mass and nitrogen uptake in plants fertilized with a mixture of the bacteria and inorganic/organic nitrogen		

↑Increased, ↓Decreased, *IAA* indole-3-acetic acid, *ACC* 1-aminocyclopropane-1-carboxylic acid deaminase, *VOCs* volatile organic compounds

*Effects on metabolic pathways and plant physiology were considered here as plant growth-promoting mechanisms

### “*Paraburkholderia busanensis*”

The most recently described species of *Paraburkholderia* is “*Paraburkholderia busanensis”*, which comprises only the strain P39 isolated from pine soil from Busan, Korea (Mannaa et al. 2023). The direct influence of this bacterium on crop growth is unknown. However, it protects green pepper fruits soaked in bacterial suspension from anthracnose caused by *Colletotrichum scovillei. “P. busanensis”* P39 produces antifungal compounds, including volatiles, which inhibit fungal growth and conidia production, thereby avoiding the development and propagation of anthracnose between pepper fruits. In addition, mycophagy caused by the activity of endochitinases was observed in *C. scovillei* (Mannaa et al. 2023). “*P. busanensis”* P39 is a promising biocontrol agent to prevent postharvest disease caused by fungi, and its potential to promote the growth of green pepper plants should be studied in more detail.

### *Paraburkholderia caledonica*

Bacteria of the species *Paraburkholderia caledonica* have been isolated from soil in countries far from each other, including the United Kingdom, Chile, and the Netherlands (Coenye et al. 2001; Carrión et al. 2018; Pérez-Moncada et al. 2024). The first attempt to demonstrate indirect PGP features was the evaluation of the role of a strain isolated from soil of sugar beet in disease-suppressive soils (Carrión et al. 2018). However, in that study, three other species of *Paraburkholderia* showed better suppression of the plant pathogen *R. solani.* Recently, a strain isolated from the Atacama Desert in Chile was included in a consortium together with yeast and arbuscular mycorrhizal fungi and demonstrated the ability to improve the growth of strawberry plants under drought stress (Pérez-Moncada et al. 2024).

This multi-inoculant significantly improved biomass (16.6%), fruit number (81.2%), K concentration (57.3%), antioxidant activity (41%), chlorophyll content (22.5%), and photosynthetic attributes. The specific influence of *P. caledonica* strains on crop growth and fitness remains unclear; however, the use of consortia to modulate crop yields is gaining attention as a more effective fertilization method in sustainable agriculture (Moretti et al. 2024).

### *Paraburkholderia oxyphila* and *Paraburkholderia eburnea*

There is limited literature on *Paraburkholderia oxyphila* and *Paraburkholderia eburnea*, and only a few strains have been described. *P. eburnea* was first isolated from peat soil in Russia (Kang et al. 2014), but non PGP traits were reported. However, the strain *P. eburnea* CS4-2 was isolated from japonica rice (*O. sativa* L. cv. Dongjin) in Korea, and has the ability to produce IAA and solubilize silicate (Kang et al. 2017). This bacterium also induced root length by up to 66%, shoot weight by up to 33% and silicon deposition in leaves of japonica rice (Kang et al. 2017), which can help alleviate biotic and abiotic stress in plants (Luyckx et al. 2017). Silicate-solubilizing bacteria are gaining attention as promising biofertilization agents for agriculture because of the importance of silicon fertilization in improving crop yields (Meena et al. 2014; Raturi et al. 2021). The first strain of *P. oxyphila* was isolated from acidic forest soil in Japan and stands out for its ability to catabolize catechin and aromatic derivatives, suggesting a role for this bacterium in the flavonoid cycle in forest soil (Otsuka et al. 2011, 2016). Recently, three strains of *P. oxyphila* named EP1, EP4, and EP5 and *P. eburnea* EP3 were isolated from black beauty eggplant (*Solanum melongena*) soil treated with rocket (*Eruca sativa*) extracts (Poulaki et al. 2024). These strains reduced the growth of the pathogen *Verticillium dahlia* on PDA plates and the disease severity and incidence in eggplant. The ability of *P. oxyphila* and *P. eburnea* to produce siderophores on M9-Cas agar, the presence of genes related to metallophores and antibiotics production, and their capacity to induce systemic resistance (Poulaki et al. 2024) suggest that these species could be effective in the biological control of *Verticillium dahliae*, a major soil-borne pathogen of global concern in recent years.

### *Paraburkholderia fungorum*

Bacteria isolated from environmental, animal, and human samples have been classified as *Paraburkholderia fungorum* (Coenye et al. 2001). The clinical significance of these bacteria remains to be fully understood, and in most cases, infections are linked to environmental sources. A relevant feature of this species is its ability to degrade aromatic hydrocarbons (Andreolli et al. 2011, 2013; Strunk and Engesser 2013; Dobslaw and Engesser 2015; Liu et al. 2019; Li et al. 2024), and bioremediation of heavy metals and ammonia (Feng et al. 2017; Liu et al. 2019). However, two environmental strains could improve crop yields.

*P. fungorum* BRRh-4, isolated from the rice rhizosphere at an experimental farm in Bangladesh (Khan et al. 2017), demonstrated PGP features in vitro, such as phosphate solubilization and IAA production, with the former being the most notable. Inoculating rice with BRRh-4 in pot experiments, conducted with soils containing zero, half, and full doses of recommended fertilizers, improved the growth and yield of the crop in two different studies (Khan et al. 2017; Islam et al. 2023). The germination percentage was modified slightly, but biomass production was significantly induced in seedling roots (25–365.38%) and aerial parts (65.71–93.13%), resulting in enhanced shoot and root length. Grain yield parameters, such as the total number of tillers per hill, number of effective tillers per hill, number of filled grains, and total grain yield per pot, also increased with the inoculation of BRRh-4. In both evaluations, using *P. fungorum* BRRh-4 combined with half the recommended dose of chemical fertilizers achieved growth and yield comparable to those obtained with the full dose (Khan et al. 2017; Islam et al. 2023). Therefore, inoculating rice with this strain could potentially reduce the need for chemical fertilizers by 50%. Additionally, positive changes in the diversity of the native bacterial communities in the rice root and rhizosphere have been reported, after the inoculation of *P. fungorum* BRRh-4, including the presence of genera such as *Prevotella*, *Bacillus,* and *Bacteroides* (Islam et al. 2023). The impact of this bacterium on the rice microbiome is crucial, as the application of PGPB-based fertilizers should not negatively affect the native microbial communities of crops.

Strawberries are an important crop worldwide owing to the increased demand for functional foods containing bioactive compounds. *P. fungorum* BRRh-4 was applied to strawberry seedling roots and foliar areas, resulting in more biomass in shoot and root (> 50%), canopy diameter, and number, length, and width of leaves (Rahman et al. 2018). Fruits are a valuable part of most crops, as they are the final products consumed. However, most studies on the PGP properties of bacteria have focused on enhancing the growth of shoots, stems, roots, and leaves. *P. fungorum* BRRh-4 has been shown to increase the total fruit weight per strawberry plant by 48% (Rahman et al. 2018). Additionally, this strain enhanced the antioxidant activities of strawberry fruits by inducing the production of antioxidant molecules, such as anthocyanins and carotenoids, with levels nearly tripling in the presence of BRRh-4 (Rahman et al. 2018). These findings suggest that *Paraburkholderia* strains could help to improve yields and produce fruits and vegetables with more nutritional and functional properties.

The most recent crop studied in combination with *P. fungorum* BRRh-4 is rapeseed (*Brassica campestris* L. cv. BARI Sarisha-14) exposed to cadmium (Raihan et al. 2022). The toxicity of this metal in plants is primarily due to oxidative stress, which occurs when the metal disrupts the electron transport chain and antioxidant defenses, leading to overproduction of reactive oxygen species (ROS) (Haider et al. 2021). The inoculation of BRRh-4 on rapeseed seeds did not affect plant growth parameters but effectively mitigated the adverse effects of cadmium exposure (Raihan et al. 2022). The presence of cadmium reduced plant height, fresh and dry root weight, and fresh and dry shoot weight. However, when rapeseed seeds were sowed in a bacterial solution and then exposed to cadmium, the height and root/shoot weight were similar to those observed in plants not exposed to the metal (Raihan et al. 2022). This strain also prevented oxidative stress induced by cadmium, as evidenced by increased antioxidant enzyme activity and reduced levels of oxidative stress markers such as malondialdehyde, hydrogen peroxide (H_2_O_2_), and electrolyte leakage (Raihan et al. 2022). As heavy metal bioremediation has previously been proposed for *P. fungorum* (Liu et al. 2019), the cadmium resistance conferred by BRRh-4 in rapeseed suggests the potential of this species to improve crop yields in contaminated soils and contribute to the bioremediation of heavy metal-contaminated soils.

Barley is another crop that has shown positive effects following inoculation with *P. fungorum* (Ibáñez et al. 2021)*.* The inoculation of barley seeds with strain PSB7, isolated from the barley rhizosphere, resulted in better phosphate accumulation in stems, greater dry weight, height of the aerial parts, and faster maturation of ears (Ibáñez et al. 2021). Direct PGP features of the strain were observed in vitro*,* including phosphate, zinc, and potassium solubilization and the production of siderophores, HCN, and IAA. Inhibition of the plant pathogens *Botrytis cinerea*, *F. oxysporum, Nigrospora oryzae*, *R. solani,* and *Alternaria alternata* has also been identified for the strain PSB7 in agar plates (Ibáñez et al. 2021). However, there is no evidence of biocontrol in plants.

Although the clinical significance of this species has not been clarified, before formulating bio-inputs containing *P. fungorum*, we consider it necessary to perform in-depth studies on the safety of each strain by phenotypic and genomic analysis because of the shared isolation of this species from human clinical samples, such as respiratory secretions, synovial tissue, urine, and duodenum tissue from HIV-1 infected patients (Coenye et al. 2002; Loong et al. 2016; Yang et al. 2016; Nally et al. 2018), and the reports of septicemia and infectious granuloma caused by *P. fungorum* (Gerrits et al. 2005; Zhang et al. 2014).

### *Paraburkholderia tropica*

*Paraburkholderia tropica* is one of the first described free-living nitrogen-fixing species of *Paraburkholderia* (Reis et al. 2004) and commonly isolated from the tissues and rhizosphere of monocots, such as maize and sugarcane (Reis et al. 2004; Kuramae et al. 2020). This species is a promising candidate for biotechnological applications such as sustainable crop growth promotion by direct and indirect PGP features, microalgal growth promotion, crude oil degradation, and antibiotic production (Tenorio-Salgado et al. 2013; Zarate et al. 2017; Bernabeu et al. 2018; Yuan et al. 2018; Sebola et al. 2019; Kuramae et al. 2020; Leite et al. 2021; Vio et al. 2022). The most common direct PGP feature of *P. tropica* strains is their ability to solubilize phosphate through organic acid production (Bernabeu et al. 2016; Kaur et al. 2016; Ghosh et al. 2019).

Although nitrogen fixation has been observed in *P. tropica* strains since their first description (Reis et al. 2004), the potential to protect crops from plant pathogens was the first PGP feature studied in depth (Tenorio-Salgado et al. 2013). Volatile compounds that displayed in vitro antifungal activity against the plant pathogens *Colletotrichum gloeosporioides*, *Fusarium culmorum*, *F. oxysporum,* and *Sclerotium rolfsii* were identified in 15 strains (Tenorio-Salgado et al. 2013). Among these bacteria, the strain MTo-431, isolated from the maize rhizosphere (Estrada-De Los Santos et al. 2001), prevented the adverse effects caused by these pathogens on maize plants growing in nutritive solution. However, to include *P. tropica* MTo-431 in bio-inputs, further greenhouse or field experiments are needed because of bacterial growth, and adaptation is easier in nutritive solutions than in soil.

The type strain of this species, Ppe8^T^, is a well-studied strain with PGP features including nitrogen fixation and IAA production (Reis et al. 2004; Chaves et al. 2015). In sugarcane plants, Ppe8^T^ induced biomass production (40–50%), nitrate reductase activity in the roots, and the content of nitrogen, potassium, and phosphorous (Gírio et al. 2015; Dos Santos et al. 2017a, b). This strain survives for over 35 days in substrates used in sugarcane propagation (Alves et al. 2023), which could lead to improved colonization of plants by bacteria and enhance crop yield. Indeed, *P. tropica* Ppe8^T^ was included in a commercial multi-inoculant composed of diazotrophic PGPBs, increasing sugarcane ratoons’ stalk and sugar yield (Kaur et al. 2016; Pereira et al. 2019).

*P. tropica* MTo-293 is an epiphyte isolated from the maize stem surface that can colonize wheat and tomato tissues (Bernabeu et al. 2015, 2018; Vio et al. 2022). This strain forms surface and endophytic populations in the roots of wheat, tomato, and tomato stems (Bernabeu et al. 2015, 2018; Vio et al. 2022). *P. tropica* produces considerable amounts of exopolysaccharide (EPS) (Serrato et al. 2006, 2008), which could act as a signaling molecule during plant-bacteria interaction and a key factor for plant colonization. The molecular mechanisms involved in the colonization of *P. tropica* MTo-293 in crops such as tomato and wheat are not well-known. However, EPS production could be an essential factor for plant infection, similar to that reported for nitrogen-fixing rhizobia in legumes (Skorupska et al. 2006). MTo-293 remains stable in potential aqueous formulations for at least 6 months (Bernabeu et al. 2018), a feature that would provide a long shelf life to bio-inputs containing this bacterium. Despite its ability to colonize dicots and monocots, positive effects on crop yields have only been observed in tomato grown in chambers and greenhouse experiments (Bernabeu et al. 2015; Vio et al. 2022). Depending on the inoculation strategy, the MTo-293 strain improved yield parameters such as the weight of fruits (inoculation of seedlings) (Bernabeu et al. 2015), roots and aerial part biomass, and total fruit (inoculation of seeds before sowing) (Vio et al. 2022). The work carried out with strain MTo-293 unveils the importance of in-deep analysis of inoculation strategies to determine the appropriate phenological stage to use *Paraburkholderia* species inoculants, as reported for other bacterial genera such as *Bacillus* (Stoll et al. 2021). The extensive study of *P. tropica* MTo-293 suggests that PGPB isolated from monocot tissues or the rhizosphere can adapt to dicot tissues and could be potent candidates for developing bio-inputs to improve their growth.

The Brazilian strain *P. tropica* IAC/BECa 135 is another example of a bacterium with high potential for bio-input use. This bacterium was isolated from sugarcane roots, produces IAA and siderophores, inhibits the growth of the plant pathogens *Fusarium verticillioides* and *Ceratocystis paradoxa* (Schlemper et al. 2018)*,* and possesses genes related to siderophores, antimicrobials, IAA production, nitrogen fixation, and phosphate solubilization (Kuramae et al. 2020). This strain also increased the biomass of sugarcane (50%) and the accumulation and use efficiency index of nitrogen (31–158%), potassium (31–164%), calcium (43–153%), magnesium (48–123%), sulphur (56–114%), iron (35–184%), manganese (82–112%), copper (60–143%) and zinc (78–142%), similar to that described above for *P. caribensis* IAC/BECa-088 (Cipriano et al. 2021; Leite et al. 2021)*. P. tropica* IAC/BECa-135 influences the rhizosphere and root endosphere microbiome (Leite et al. 2021), supporting the idea that more studies should analyze the plant–microbe interactions of *Paraburkholderia* strains from the holobiont perspective. IAC/BECa 135 has also been studied in sugarcane plantlets grown under high aluminum saturation (Labanca et al. 2020). After inoculation with IAC/BECa 135, plantlets showed higher shoot biomass and calcium and boron contents. The uptake of calcium and boron can prevent physiological processes affected by oxidative damage caused by toxic levels of aluminum in the soil (Labanca et al. 2020). Then, this strain could be used for bioremediation purposes and to increase the growth of crops in metal-saturated soils.

Another monocot suitable for use with *P. tropica* is sorghum. Inoculation with strain IAC/BECa 135 enhanced biomass production in this crop (Schlemper et al. 2018). However, this effect is cultivar-dependent (Schlemper et al. 2018), and little is known about the molecular interaction with this crop, which restricts its effective use.

The extensive research on *P. tropica* over the last decade has shown the potential of this species to achieve sustainable agricultural practices to improve crop yields using bacteria from the genus *Paraburkholderia*, especially in monocots such as sugarcane and maize. Regarding this issue, we highlight the successful use of this species in commercial inoculants in Brazil.

### *Paraburkholderia* sp.

Some strains of *Paraburkholderia* identified only at the genus level also have the potential to increase crop yield through direct and indirect mechanisms, including the bioncontrol of fungal pathogens. We will describe these cases next.

The strain NhPBG1, isolated from the carnivorous plant *Nepenthes hamblack* revealed direct PGP properties such as IAA and ammonia production, phosphate, potassium, zinc solubilization, ACC deaminase, and nitrogen fixation activity (Ravi et al. 2021). This strain produces different putative antimicrobial compounds and inhibits the growth of the plant pathogens *Pythium aphanidermatum*, *R. solani*, *F. oxysporum,* and *Colletotrichum accutatum* on agar plates and the colonization of *P. aphanidermatum* in ginger rhizomes. These results suggest the potential of NhPBG1 to act as a PGPB and biocontrol agent.

*Paraburkholderia* sp. B25 was isolated from the rhizosphere of maize in Marne, France (Backes et al. 2021b). This strain restricted the growth of *Drechslera teres* fungus on PDA plates and in detached barley leaf assays (Backes et al. 2021a, b). *D. teres* is the causal agent of net blotch, which is one of the most important diseases related to barley yield loss. The disease affects the photosynthetic performance of barley leaves; however, this effect decreases when B25 is inoculated into the leaves before fungal spores (Backes et al. 2021b). B25 did not modify metabolite production or plant defense gene expression (Backes et al. 2021a). Thus, its protective effects are probably derived from the production of antifungal molecules.

In Brazil, *Paraburkholderia* sp. ESA 688 and ESA 700 were isolated from *Vigna radiata* nodules, a plant used as a trap host to isolate microorganisms from soil samples from the dry forest of pristine Caatinga (da Silva et al. 2021). Both strains increased the percentage of cowpea (*Vigna unguiculata* L. Walp.) seed emergence (40%) and showed slight efficiency (22 and 30%) in controlling the growth of *Fusarium* sp. Antimicrobial activity is rarely observed in nodulating *Paraburkholderia.* Thus, the inhibition of fungal plant pathogens suggests the potential of strains ESA 688 and ESA 700 to improve legume health by enhancing nitrogen uptake and controlling the growth of plant pathogens.

*Paraburkholderia* sp. P12 and P17 isolated from the blueberry rhizosphere increased the seed germination rate of blueberry and showed in vitro inorganic phosphate solubilization, auxin production, and nitrogen fixation (Wang et al. 2022). Interestingly, strain P17 also solubilized silicate. Insoluble silicates are a source of silicon, which has beneficial roles in vascular plants, including improving plant fitness under biotic and abiotic stress (Raturi et al. 2021). Silicate solubilization is not a commonly studied PGP feature of bacteria proposed to enhance crop yields, and *Paraburkholderia* sp. P17, together with the previously described *P. eburnea* CS4-2, opens the door for a detailed study of this ability in the genus and the positive effects of solubilized silicate in plants.

*Paraburkholderia* sp. SOS3 is a bacterium used in a formulation based on PGPR (Sustainable Organic Solutions Pty Ltd; NCBI ID: 1,926,494), with beneficial effects on the growth of different plants, such as the therapeutic honey plant *Leptospermum polygalifolum* Salisb.*,* kikuyu grass (*Pennisetum clandestinum* Hochst. Ex Chiov), *Macadamia integrifolia* Maiden and Betch, sugarcane, sorghum, avocado, and rice (Paungfoo-Lonhienne et al. 2019, 2020, 2021; Gallart et al. 2021, 2022; Kanjanasopa et al. 2021; Petersen et al. 2021; Grunennvaldt et al. 2022). Studies on this strain have included features such as compatibility with chemical fertilizers and culture conditions, which are two important aspects during inoculant development.

Strain SOS3 improved the growth parameters of sugarcane fertilized with organic and inorganic products in experiments conducted in glasshouses. These parameters included weight (22–33%), height, sett number(13–36%), tiller number per hectare (20%), and sucrose yield (46%) (Paungfoo-Lonhienne et al. 2021, 2020). In macadamia seeds, this strain promoted total biomass (32%), nitrogen (43%), and potassium (43%) uptake and reduced mineral nitrogen leaching when inoculated with inorganic and organic nitrogen (Gallart et al. 2021). Recently, nitrogen uptake (23%) and nitrogen biomass in the rhizosphere (100%) were improved in reed avocado inoculated with SOS3 and organic/inorganic fertilizers (Gallart et al. 2022). PGP properties were observed in seedlings fertilized with inorganic or organic/inorganic fertilizers. The strain did not affect plants fertilized with inorganic products, similar to that previously observed in macadamia (Gallart et al. 2021). Owing to the increasing popularity of avocado worldwide, it is essential to prevent the negative environmental impacts of its intensive production. The use of PGPR is a suitable strategy to reduce the use of chemical fertilizers and their ecological implications. The SOS3 studies on sugarcane, macadamia, and avocado demonstrated that PGPB of the genus *Paraburkholderia* could be combined with reduced chemical fertilizer doses to improve the yield of these crops.

*Paraburkholderia* sp. SOS3 also modified the percentage of emergence, biomass, height (18–19%), and vigor index (50%) in sorghum and maize plants (Petersen et al. 2021). This effect was enhanced when glucose was added to batch cultures of SOS3 (Petersen et al. 2021). Proteomic analysis of this glucose-supplemented medium showed a significant abundance of enzymes involved in the biosynthesis of molecules related to PGP properties such as nitrogen fixation, phosphate solubilization, phytohormone production, and siderophore production, suggesting that the biomass obtained from these cultures is biochemically competent to promote plant growth after seed inoculation (Petersen et al. 2021). The study of the effect of carbon sources on *Paraburkholderia* sp. SOS3 growth and PGP features revealed the importance of culture conditions in obtaining a suitable biomass of *Paraburkholderia* strains for bioinoculants to improve crop yield.

Regarding biocontrol features, SOS3 decreased the growth of the fungal pathogens *Fusarium moniliforme*, *Helminthosporium oryzae, Pyricularia oryzae,* and *Curvularia lunata* on rice seeds soaked in this bacterium and grown on water agar and PDA plates (Kanjanasopa et al. 2021). However, this protection disappeared in seedlings, indicating that SOS3 could only be used to protect seeds from pathogens (Kanjanasopa et al. 2021).

## Species for growth promotion and biocontrol of bacterial and fungal diseases

Besides the direct effects on the growth of different crops, some *Paraburkholderia* species have shown the potential to control diseases caused by different types of plant pathogens, such as bacteria and fungi (Table 4). These species are described below.

**Table 4:** *Paraburkholderia* species with the potential to promote crop growth and control bacterial and fungal diseases

*Paraburkholderia* species	Strain (s)	Source	Tested crop	Effects on crop growth/yield	Plant growth-promoting mechanism(s) reported in the species (Direct and indirect) *	Reference
*P. bryophila*	A2, A5, A17, A20, A22, 1A11, 1A16, 1S5, 1S18T, 2Sp58 and 2Sp83	Red peat moss (*Sphagnum rubellum* Wilson.)	Lettuce (*Lactuca sativa* L.)	↑ Plant, root and leaf length	Siderophore production, ACC activity and in vitro inhibition of plant pathogens (*Verticillium dahliae, Rhizoctonia solani, Xanthomonas campestris* and *Pythium ultimum*)	Vandamme et al. (2007)
*P. graminis*	PSH1	Sugar beet (*Beta vulgaris* L. subsp. *vulgaris*) soil	Sugar beet (*B. vulgaris* subsp. *vulgaris*)	Suppression of *Rhizoctonia* damping-off	Production of sulfurous VOC’s with antifungal activity Changes in soluble sugars generation and utilization Induction of the accumulation of secondary metabolites related to induced resistance Activation of carbohydrate use by primary pathways Induction of the expression/production of the nitrate transporter NAR2 Induction of systemic resistance to pathogens when co-inoculated with the fungus *Funneliformis mosseae*	Carrión et al. (2018)
			Broccoli (*Brassica oleracea* var. Italica Plenck)	↑ Root and shoot biomass ↓ Disease severity of two *X. campestris* pathovars in broccoli leaves		Jeon et al. (2021)
	C4D1M^T^	Maize (*Zea mays* L.) roots	Wheat (*Triticum aestivum* cv. Chinese Spring)	↑ Seed production and spike weight ↓ Disease symptoms caused by *Xanthomonas translucens*		Vannini et al. (2021)
*P. hospita*	mHSR1	Sugar beet (*B. vulgaris* subsp. *vulgaris*) soil	Sugar beet (*B. vulgaris* subsp. *vulgaris*)	Suppression of *Rhizoctonia* damping-off	Changes in soluble sugars generation and utilization Induction of the accumulation of secondary metabolites related to induced resistance	Carrión et al. (2018)
			Broccoli (*B. oleracea* var. Italica)	↑ Root and shoot biomass ↓ Disease severity of two *X. campestris* pathovars in broccoli leaves		Jeon et al. (2021)
*P. megapolitana*	A1, A3 and A10	Bog groove moss (*Aulacomnium palustre* Hedw. Schwärg.)	Lettuce (*L. sativa*)	↑ Plant, root and leaf length	Siderophore production and in vitro inhibition of plant pathogens (*V. dahliae, R. solani and X. campestris*)	Vandamme et al. (2007)
*P. phytofirmans*	PsJN^T^	Onion (*Allium cepa* L.) roots	Potato planlets (*Solanum tuberosum* L.)	↑ Root number, root dry and fresh weight, halum dry weight, stem length, shoot length and fresh weight ↑ Leaf hair formation, secondary branching and total plant lignin content	Inhibition the plant pathogen *Botrytis. cinerea* by direct antimicrobial effect, inhibition of spore germination, biofilm formation, priming plant innate immunity (H_2_O_2_ production, callose deposition, induction of salicylic and jasmonic acid genes) and modulation of leaf carbohydrate metabolism In vivo control of Pierce’s Disease (*Xylella fastidiosa*) by induction of the expression of innate disease resistance genes, and alterations in the metabolome of the pathogen related to host adaptation and virulence Control of trunk disease caused by *Neofusicoccum parvum* via up regulating the expression of plant defense genes Induction of the expression of genes encoding proteins with antifreeze and antifungal activities and genes implicated in plant stress responses (resveratrol, salicylic acid and jasmonic acid production) Increased levels of stress-related metabolites (proline, H_2_O_2_ and aldehydes) Increased carbohydrates involved in cold stress tolerance Induced changes in the expression of genes related to cytosine methylation, growth, stress response, hormone metabolism, signal transduction, disease resistance and phenolic compounds production Induction of changes in the microbiome	Frommel et al. (1991), Da et al. (2012)
			Wheat (*Triticum aesvitum* cv. Saher)	↑ Photosynthetic and transpiration rates, stomatal conductance, substomatal CO_2_ content, straw yield, grain weight, grain yield, antioxidant defenses, and mineral nutrition		Naveed et al. (2014)
			Tomato (*L. esculentum* Mill.)	↑ Shoot and root dry weight and shoot length and phenolic compounds ↑ Verticillium wilt resistance ↑ Fresh weight of plant at 25 and 32ºC ↑ Chlorophyll content and gas exchange at 25 and 32ºC ↑ Photosynthesis net rate at 32ºC ↑ Sugar accumulation. Increased shoot length after the strain transmission by mirids ↓ Lipid peroxidation after cold stress exposure		Pillay and Nowak (1997), Sharma and Nowak (1998), Issa et al. (2018), Galambos et al. (2020, 2021), Licciardello et al. (2024)
			Grapevine planlets (*Vitis vinifera* L.)	↑ Root growth and planlets biomass, secondary roots, root and leaf hairs ↑ Shoot and root dry weight, number of nodes, and CO_2_ fixation ↓ Susceptibility to gray mold disease ↑ Tolerance to cold stress, production of anthocyanins, tannins and flavonoids		Ait Barka et al. (2000), Fernandez et al. (2012), Theocharis et al*.* (2012), Miotto-Vilanova et al. (2016), Baccari et al. (2019), Miotto-Vilanova et al. (2019), Wu et al. (2020), Vilanova et al. (2022), Feitosa-Junior et al. (2024)
			Switchgrass (*Panicum virgatum* L.)	↑ Fresh and dry weight, leaf area, height, overground, shoot, root and total biomass ↑ Photosynthetic rates and water use efficiency		Kim et al. (2012), Wang et al. (2015)
			Spring wheat (*Triticum aestivum* L.)	Colonized seed showed earlier spike onset		Mitter et al. (2017)
			Rapeseed (*Brassica napus* L.)	↑ Plant height, root length, fresh/dry shoot and root biomass in plants growing in chromium-contaminated soils ↑ Photosynthetic and transpiration rates, stomatal conductance, chlorophyll contents, substomatal CO_2_ concentration and water use efficiency ↓ Chromium uptake by plants		Nafees et al. (2018)
			Quinoa (*Chenopodium quinoa* Willd.)	↓ Effects of salinity on plant height, shoot biomass panicle length, grains weight and grain yield per plant		Yang et al. (2020)
			Rice (*Oryza sativa* L.)	↑ Root biomass		King et al. (2023)
*P. terricola*	mHS1	Sugar beet (*B. vulgaris* subsp. *vulgaris*) soil	Sugar beet (*B. vulgaris* subsp. *vulgaris*)	Suppression of *Rhizoctonia* damping-off	Changes in soluble sugars generation and utilization Induction of the accumulation of secondary metabolites related to induced resistance	Carrión et al. (2018)
			Broccoli (*B. oleracea* var. Italica)	↑ Root and shoot biomass ↓ Disease severity of two *X. campestris* pathovars in broccoli leaves		Jeon et al. (2021)

↑Increased, ↓Decreased, *IAA* indole-3-acetic acid, *ACC* 1-aminocyclopropane-1-carboxylic acid deaminase, *VOCs* volatile organic compounds

*Effects on gene expression, metabolic pathways, and plant physiology were considered plant growth-promoting mechanisms

### *Paraburkholderia megapolitana* and *Paraburkholderia bryophila*

*Paraburkholderia megapolitana* (three strains) and *Paraburkholderia bryophila* (11 strains) were first described in 2007 (Vandamme et al. 2007) and include bacteria isolated from mosses, woodland roots, and rotting wood. Both species have demonstrated some PGP properties in vitro and promoted the overall length of lettuce plants (31%), including a 36% increase in root length and a 57% increase in leaf length. The production of siderophores was the only direct PGP mechanism determined in vitro across all the strains of both species. However, some strains of *P. bryophila* also exhibit ACC deaminase activity.

Antagonistic activity against *V. dahliae*, the causal agent of verticillium wilt in many plant species, was identified as an indirect PGP mechanism. Additionally, some strains of *P. megapolitana* and *P. bryophila* inhibited the growth of the phytopathogens *R. solani*, *Erwinia carotovorans,* and *P. ultimum*, indicating their potential as PGPB and biocontrol agents. The antagonistic strains of *P. megapolitana* produced lytic enzymes, such as proteases and β-glucanases, which are probably related to their antimicrobial activity. Furthermore, some strains were antagonistic against *Candida albicans* and *Staphylococcus aureus*, suggesting their use as a source of antimicrobials for human pathogens.

This report continues as a solo study on the potential of *P. megapolitana* and *P. bryophila* to promote plant growth. Despite their ability to inhibit a range of plant pathogens, including bacteria, fungi, and oomycetes, no additional studies have been published to date. We consider both species as promising for biocontrol applications, and further research in plants is necessary to explore and confirm their efficacy.

### *Paraburkholderia graminis*, *Paraburkholderia hospita* and *Paraburkholderia terricola*

*Paraburkholderia graminis* is a species commonly isolated from grasses soil or tissues (*Poaceae* family) (Viallard et al. 1998; Castanheira et al. 2016; Kandasamy et al. 2019). In recent years, strains of this species have emerged as promising enzyme-producing bacteria with potential applications in the food and pharmaceutical industries (Mathew et al. 2015; Fan et al. 2017; Tang et al. 2022; Zhang et al. 2022; Bahlawan et al. 2023). However, their potential to improve plant growth is also known in wheat and non-crop plants, such as *A. thaliana* and ryegrass (*Lolium multiflorum* Lam.) (Castanheira et al. 2016; Duan et al. 2023). Three strains isolated from corn induced changes in shoot length and root weight after co-inoculation of wheat plants (Kandasamy et al. 2019). The maize-associated C4D1M^T^ increased seed production in adult plants and duplicated the spike weight of wheat (Vannini et al. 2021). When co-inoculated with the arbuscular mycorrhizal fungus (AMF) *Funneliformis mosseae,* a reduction in the disease caused by *Xanthomonas translucens,* a leaf pathogen in wheat, was observed (Vannini et al. 2021). This effect is derived from the induction of the expression of proteins related to systemic resistance in the leaves. Inoculation with C4D1M^T^ also affected sugar metabolism and enhanced the concentration of nitrogen and amino acids in plants. Both studies suggest the potential of *P. graminis* to improve wheat yields, and further studies should be performed on other grasses from the *Poaceae* family to explore this species as bio-inputs for crops of this family.

A particular case of *P. graminis* isolated from no-grass plants is the strain PSH1. This bacterium was isolated together with *Paraburkholderia hospita* mHSR1 and *Paraburkholderia terricola* mHS1 from suppressive soils in a sugar beet field in The Netherlands (Carrión et al. 2018). Suppressive soils are essential sources of antagonistic bacteria that can act as PGPB, and *P. graminis* PSH1 has shown in vitro antagonistic activity against *R. solani* due to the production of sulphurous volatile organic compounds (VOCs)*.* This fungal root pathogen causes damping-off in sugar beet. However, in vivo*,* this disease was only suppressed by *P. hospita* mHSR1 and *P. terricola* mHS1. In another study, after root tip inoculation, the three species colonized the roots and boosted the root (50–250%) and shoot (70–100%) biomass of broccoli (Jeon et al. 2021). The three species also changed soluble sugar generation and utilization in the shoots, which could be related to the production of sufficient energy to promote increased plant growth. In addition, these species induced an accumulation of secondary metabolites related to immunity against the disease caused by two pathovars of *Xanthomonas campestris* in the leaves (Jeon et al. 2021). Soil from sugar beet could be considered a source of *P. terricola* isolation, as other strains were also found in the rhizosphere of this crop in Belgium (Gasser et al. 2009). Interestingly, *P. hospita and P. terricola* were isolated simultaneously in previous research (Goris et al. 2002) but are still poorly studied species, and no more reports propose their application to improve crop yields. However, *P. graminis* is a promising species that can elevate wheat and broccoli yields and control bacterial diseases caused by *Xanthomonas* pathogens in both crops.

### *Paraburkholderia phytofirmans*

*Paraburkholderia phytofirmans* is the most studied species of *Paraburkholderia* regarding PGP activities. The type strain PsJN^T^ is an endophytic bacterium (Compant et al. 2005, 2008) that has been studied for over 30 years and has been shown to promote plant growth under in vitro, growth chamber, and greenhouse conditions. The review published by Esmaeel et al. (2018) provides detailed information on the perception, colonization, distribution, PGP, and protective abilities of the species-type strain.

PsJN^T^ has been reported to promote plant biomass in plants such as potato, tomato, grapevine, and rice. In potato, this strain increased root (157%) and stem (176%) biomass, leaf hairs (55–110%), secondary root formation, and lignin content (43%) (Frommel et al. 1991; Da et al. 2012). Positive effects on tomato include higher production of root biomass (69%), height (27–300%) and phenolic compounds (Pillay and Nowak 1997; Sharma and Nowak 1998; Galambos et al. 2020, 2021; Licciardello et al. 2024), as well as a reduction in lipid peroxidation after cold stress exposure (Licciardello et al. 2024), suggesting the potential of this strain to help mitigate abiotic stress in plants. The strain can be transmitted between tomato plants by the beneficial mirid species *Macrolophus pygmaeus* and *Nesidiocoris tenuis,* increasing the height of the recipient plants (Galambos et al. 2021). Furthermore, inoculation with PsJN^T^ complemented with humic acid enhanced tomato growth and induced the expression of genes related to signal transduction, hormone metabolism, defense, and plant growth (Galambos et al. 2020).

The root biomass of rice was boosted after inoculation with *P. phytofirmans* PsJN^T^, which quickly colonizes the root endosphere and induces changes in the expression patterns of genes related to plant immunity (King et al. 2023). Modulation of immune genes could be significant for developing resistance to plant pathogens that affect rice yield, even when this bacterium does not directly induce the growth of this crop.

Grapevine is another crop with substantial evidence of the growth-promoting effects of PsJN^T^. In this crop, inoculation with PsJN^T^ resulted in improved shoot (239%), root (213–1100%) and total (225–500%) biomass, among other parameters (Barka et al. 2000; Ait Barka et al. 2006). PsJN^T^ can also be transmitted as a seed endophyte and improve the crop yield of daughter plants. When the strain was sprayed on wheat, maize, pepper, and soybean flowers, the seeds produced by these flowers showed an earlier spike onset in greenhouse and field experiments, and the number of ears per square meter was higher in the colonized seeds (Mitter et al. 2017). Furthermore, changes in the microbiome of plants from colonized seeds were observed, increasing the abundance of β-Proteobacteria and Flavobacteria and reducing α-Proteobacteria.

*P. phytofirmans* PsJN^T^ can also promote crop plant growth under abiotic stress such as drought, cold, high temperature, high salinity, and heavy metals contamination. Drought stress affects the growth, physiology, and yield of crops, including wheat (Qaseem et al. 2019). In this crop, drought induces negative changes in the photosynthetic and transpiration rates, stomatal conductance, substomatal CO_2_ content, straw yield, grain weight, and grain yield of wheat, but *P. phytofirmans* PsJN^T^ can prevent this damage (Naveed et al. 2014). This abiotic stress induces the production of ROS, which stimulates enzymatic and non-enzymatic antioxidant defenses. The protective response to ROS was induced in wheat after inoculation with PsJN^T^ (Naveed et al. 2014). This bacterium also benefits the cold acclimatization of grapevine plantlets by modifying carbohydrate metabolism, leading to an accumulation of carbohydrates, such as glucose, which are involved in the tolerance to cold stress (Fernandez et al. 2012). Additionally, in this crop, PsJN^T^ improved secondary metabolite production, highlighting the production of anthocyanins, tannins, and flavonoids, some of which have been suggested to participate in the response to the plant pathogen *B. cinerea* (Miotto-Vilanova et al. 2019)*.* Furthermore, the impact of PsJN^T^ on the induction of high-temperature tolerance has been studied in tomato (Issa et al. 2018). The strain showed rapid colonization of the rhizoplane and the interior parts of the roots and stems of tomato plants and increased the fresh weight of plants. The growth of quinoa plants was affected by high salinity in greenhouse experiments; however, when quinoa seeds were inoculated with *P. phytofirmans* PsJN^T^ before sown, the negative effect of salinity on plant height, shoot biomass, panicle length, grain weight, and grain yield per plant was decreased (Yang et al. 2020). This study suggests that this bacterium has a positive effect on plant growth in high-salinity soils.

The growth and physiology of rapeseeds (*Brassica napus* L.) grown in chromium-contaminated soils were also stimulated after inoculation with PsJN^T^ mixed with biogas slurry (BGS) (Nafees et al. 2018). This effect included enhanced root (42–101%) and shoot (23–95%) biomass, photosynthetic rate (42–46%), stomatal conductance (81%-93%), chlorophyll contents (36–51%) and sub-stomatal CO_2_ concentration (53–54%). The treatment also reduced plant chromium uptake, suggesting the potential of this bacterium to decrease heavy metal bioaccumulation in plants.

An indirect mechanism studied in *P. phytofirmans* PsJN^T^ to increase crop yields is its use as a biocontrol agent. The strain protects plants from verticillium wilt during early growth by inducing the host defensive response, resulting in taller plants even in the presence of the pathogen (Sharma and Nowak 1998). PsJN^T^ showed a direct antimicrobial effect against *B. cinerea* by forming biofilm around the fungal mycelium in grapevine leaves, stimulating plant innate immunity (Miotto-Vilanova et al. 2016), putative direct antimicrobial effect, and inhibiting spore germination (Vilanova et al. 2022). *P. phytofirmans* PsJN^T^ can also induce a priming effect on plant immunity, reducing the severity of Pierce’s disease caused by *Xylella fastidiosa* in grapevine in greenhouse and field experiments (Baccari et al. 2019; Lindow et al. 2023). PsJN^T^ modifies the production of metabolites related to the host adaptation and virulence of *X. fastidiosa* and the structure of the biofilm produced by this pathogen, which represents an important virulence factor (Feitosa-Junior et al. 2024). These effects confirm the potential of PsJN^T^ to control *X. fastidiosa* in grapevine farmers. Additionally, despite the effectiveness of *P. phytofirmas* PsJN^T^ in preventing plant pathogens by itself, trunk disease of grapevine caused by *Neofusicoccum parvum* could be controlled better with this strain when inoculated with the fungicide fenpiclonil (Wu et al. 2020). The ability of PGPB to control plant diseases by itself is challenging to achieve; however, they could be considered for integrated control strategies (ICS) when their use reduces the doses of agrochemicals required to control plant diseases. The biological control of *N. parvum* with PsJN^T^ and fenpiclonil confirmed that this bacterium could be used in ICS.

An unexplored topic of *Paraburkholderia* species is their impact on plant epigenetics. However, interesting effects of PsJN^T^ were observed on cytosine methylation in potato (Da et al. 2012). Induced methylation polymorphisms were observed in genes related to signal transduction, stress response, protein synthesis/degradation, and cell wall structure. The expression levels of genes involved in growth, stress response, and disease resistance increased in bacterized plants, whereas the expression of senescence-related genes decreased. Disease-resistance genes were only related to plant recovery after endophyte infection. However, this could be explored in biocontrol tests to determine whether PsJN^T^ induces resistance to plant pathogens.

The extensive evidence of the positive influence of *P. phytofirmans* PsJN^T^ on crop yield through direct and indirect PGP mechanisms clearly shows that researchers should pay more attention to *Paraburkholderia* species and their potential for developing bioinoculants.

## Conclusions

In the coming decades, agriculture will face the challenge of producing sufficient food and consolidating global food security. The impact of climate change on plant growth, the loss of soil fertility, and the contamination of agricultural soils will lead scientists to look for strategies and develop sustainable technologies that allow the consolidation of food security. Plant-associated bacteria have been studied and proposed as alternatives to chemical fertilizers and pesticides for decades because of their beneficial effects on plant growth, including most plants used as crops to generate food. The genus *Paraburkholderia* comprises at least 24 species that promote the growth of crops of agricultural interest, which can significantly impact their yields. The PGP mechanisms reported in this genus include regulation of plant growth by auxin production and ACC deaminase enzyme activity, enhancement of nutrient acquisition mediated by siderophore production, phosphate solubilization, nitrogen fixation, and prevention of yield losses due to diseases caused by pathogenic microorganisms through the induction of systemic immunity and production of antimicrobial compounds. Likewise, bacteria of the genus *Paraburkholderia* stimulate the expression of genes related to the production, processing, and transport of macromolecules, organ development, physiological processes such as photosynthesis, and the activity of antioxidant defenses. Some strains improve plant growth under abiotic stress conditions such as salinity, drought, or heavy metal contamination. These bacteria can modify the crop microbiome, which has recently become extremely important for optimal plant growth and development. Most of these results have been obtained in laboratory or greenhouse experiments; therefore, increasing the number of experiments in the field is necessary. However, the evidence compiled in this review indicates the potential of the genus *Paraburkholderia* for developing products that reduce the use of chemical fertilizers and pesticides, whose adverse effects on the environment and human and animal health are widely known.

## Data Availability

No datasets were generated or analysed during the current study.
